# Revisiting the Fertility Transition in England and Wales: The Role of Social Class and Migration

**DOI:** 10.1007/s13524-020-00895-3

**Published:** 2020-07-01

**Authors:** Hannaliis Jaadla, Alice Reid, Eilidh Garrett, Kevin Schürer, Joseph Day

**Affiliations:** 1 Cambridge Group for the History of Population and Social Structure, Department of Geography, University of Cambridge, Cambridge, CB2 3EN UK; 2 Estonian Institute for Population Studies, Tallinn University, 10120 Tallinn, Estonia; 3 Department of History, University of Essex, Colchester, CO4 3SQ UK; 4 Centre for English Local History, University of Leicester, Leicester, LE1 7QR England

**Keywords:** Fertility transition, Nineteenth century, Census microdata, Migration, England and Wales

## Abstract

We use individual-level census data for England and Wales for the period 1851–1911 to investigate the interplay between social class and geographical context determining patterns of childbearing during the fertility transition. We also consider the effect of spatial mobility or lifetime migration on individual fertility behavior in the early phases of demographic modernization. Prior research on the fertility transition in England and Wales has demonstrated substantial variation in fertility levels and declines by different social groups; however, these findings were generally reported at a broad geographical level, disguising local variation and complicated by residential segregation along social class and occupational lines. Our findings confirm a clear pattern of widening social class differences in recent net fertility, providing strong support for the argument that belonging to a certain social group was an important determinant of early adoption of new reproductive behavior in marriage in England and Wales. However, a relatively constant effect of lower net fertility among long-distance migrants both before the transition and in the early phases of declining fertility indicates that life course migration patterns were most likely factor in explaining the differences in fertility operating through postponement of marriage and childbearing.

**Supplementary Information:**

The online version contains supplementary material available at 10.1007/s13524-020-00895-3.

## Introduction

The vast body of research on the determinants of the historical fertility transition has generally located its origins at the time, in the late nineteenth and early twentieth century, when contemporaries first started noticing profound changes in fertility in their society. A century or more later, we have a far more complete understanding of the timing of the process and overall patterns of decline, but the precise causal pathways that led to reduced fertility remain somewhat speculative and subject to debate (Becker 1981; Cleland and Wilson 1987; Coale and Watkins 1986; Easterlin 1975; Galor and Weil 2000; Mason 1997). A particular research challenge has been to formulate an explanatory framework of fertility decline that can account for the full diversity of experiences from high to low fertility across an array of distinct social and economic contexts.

Broadly defined, explanations of fertility decline often distinguish between two concepts of behavioral change—innovation and adjustment—that need to occur before new reproductive habits are adopted (Carlsson 1966). The innovation perspective attributes falling fertility to the spread of new knowledge of means of contraception and attitudes, and the adjustment or adaptation perspective conceptualizes fertility decline as a response to a transformation of the economic and social environment. The latter perspective is closely related to the associated changes in the costs of having children and to the concept of the demand and supply of children (Easterlin and Crimmins 1985). The main evidence for this argument outlines the changes in economic organization during the nineteenth century, when the introduction of restrictions on children’s participation in the labor force and the enforcement of school attendance combined to increase the relative costs of having large families.

Adherents to the two forms of explanation generally agree that analysis of the extent of socioeconomic variations in fertility and how these are transformed during the process of fertility decline is fundamental to any understanding of the nature of fertility transition. Previous research has highlighted that socioeconomic differences in fertility tend to widen as fertility transitions begin and that the upper and middle classes were most often the first to move toward lower fertility (Dribe and Scalone 2014; Dribe et al. 2014; Haines 1992). The general view from previous work is that both adjustment and innovation processes lie behind the observed socioeconomic patterns. It is thought that higher social groups were more likely to adapt their fertility behavior to new economic circumstances; they were also thought to be the first group of parents to seek fewer, higher-quality children rather than a higher quantity of offspring to match their material aspirations (Dribe 2009). In addition, fitting with the innovation perspective, the upper and middle classes were the first to develop new social attitudes toward family planning because they were in a better position to acquire new knowledge and information through education and through their social networks, which extended over wide distances (Szreter 1996; Woods 1987).

Access to information has an important role in many of the debates about the origins of historical fertility transition. Behavioral changes in society are seen as the result of new ideas and values spreading through the population; this spread of information was, however, limited by spatial and social distance (Garrett et al. 2001; Goldstein and Klüsener 2014; Szreter 1996). The increasing spatial mobility and rapid urbanization of the population, especially during the latter half of the nineteenth century, made an important contribution to this process. Clearly, relocation to a new social environment often went hand in hand with important life course events. Based on previous research, migration is often seen to influence an individual’s fertility behavior through four possible mechanisms: socialization, adaptation, selection, and disruption (Kulu 2005).

Recently, Klüsener et al. (2019) argued that lifetime migration and distances migrated could be indicative of the spread of information and the expansion of knowledge networks, leading to the adoption of fertility limitation. This tenet is fundamentally based on two assumptions. First, migrants living farther from their birthplace might have had better access to information because their social networks covered larger areas and longer distances. Second, recent migrants to an urban settlement may have found it easier to adopt new social attitudes to family limitation as a direct result of finding themselves free of the tighter social controls of their native villages. The fertility decisions of city dwellers were possibly less influenced by the pressures of family, members of the older generation, and their home community to have larger families. Migration may also be correlated to fertility through co-determination by an unmeasured variable: migrants are likely to be selective of the more enterprising, ambitious, and open-to-innovation among members of the community they leave (Creighton et al. 2012). These characteristics may produce both longer-distance migration and a willingness to adopt new fertility-controlling strategies. However, the disruptive nature of migration, coupled with the difficulties of integrating into a new environment, may have also left migrants disinclined to have large families, particularly when they had no local or familial support networks in their new place of residence (Creighton et al. 2012).

Alternatively, the relationship could be the result of reverse causality. Previous evidence has suggested that in the British Isles, most lifetime migration occurred in the young adult period, before or at marriage, and generally before the birth of children (Day 2015, 2018a; Reid et al. 2016; Schürer 2003; Wall 1987). It is likely that early marriage and childbearing are a strong disincentive to migration: they make moving logistically more problematic and also more costly such that individuals who choose (or are forced) to start a family young may be less likely to migrate. For example, from the perspective of place of destination, migrants to Antwerp (Belgium) and Geneva (Switzerland) were more likely to postpone marriage and childbearing to a later age than the native populations. In Antwerp, this was particularly clear among long-distance migrants (Schumacher et al. 2013). However, an analysis of fertility behavior of leavers and stayers from the perspective of their place of origin might reveal even wider differences. Over the course of the nineteenth century, the magnitude and context of geographical mobility changed dramatically. This was a period of rapid modernization, including the remarkably fast development of transport networks. In particular, the expansion of the railways meant that previous ideas of what constituted short and long distances were being transformed—an additional aspect of the changes underway in society (Gregory and Henneberg 2010).

## The Context: Fertility Transition in England and Wales

**Fig. 1:**
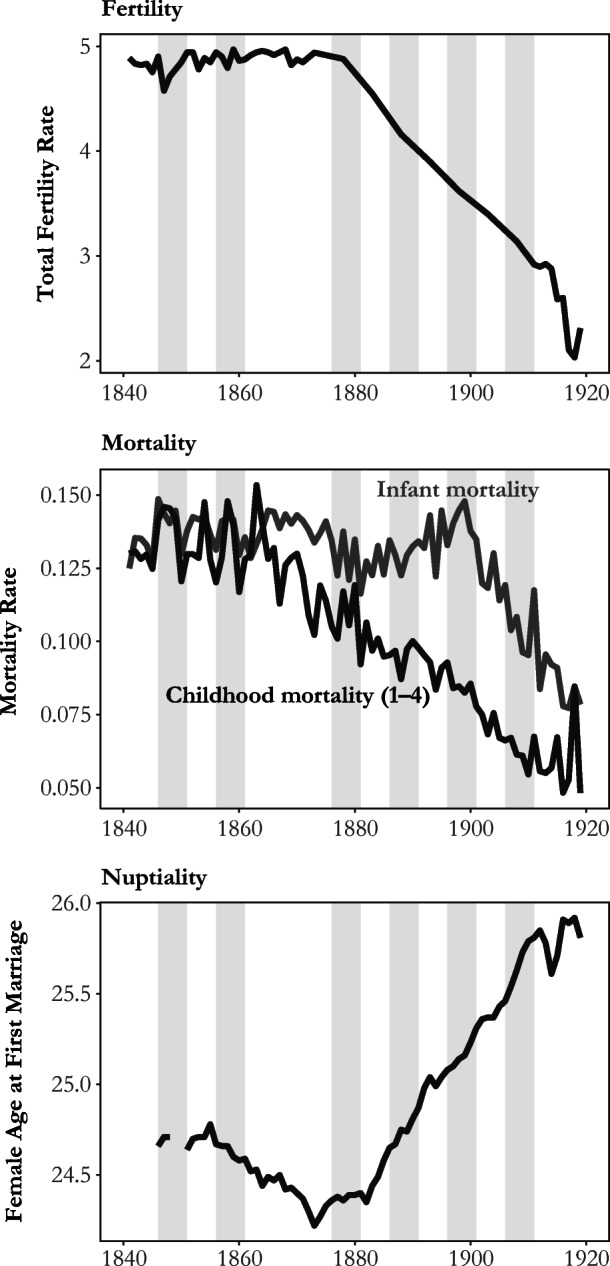
Long-term trends in fertility, infant and childhood mortality, and nuptiality in England and Wales, 1840–1920. Shaded bands show the five years before each census*. Source:* Woods (2000); Human Mortality Database (2019); Wrigley et al. (1997:134); Office of National Statistics (2011).

In this study, we aim to revisit the debate on fertility transition by using rich individual-level decennial census data for England and Wales (1851–1911) to investigate the effects of social class and spatial mobility on individual fertility behavior. The countries’ early industrialization and urbanization, geographically clustered industries, and complex occupational structure all provide an ideal setting in which to test the influence of class and increasing population mobility on fertility decline. Figure 1 highlights the dramatic changes in fertility, mortality, and nuptiality that England and Wales experienced between the 1840s and the 1920s.

Research on the fertility transition in the British Isles has established that a substantial fall in marital fertility took place over the second half of the nineteenth century (Coale and Watkins 1986; Morse 1987; Teitelbaum 1984; Woods 1979, 2000). Studies have also found substantial variation in fertility levels and rates of decline between social groups (Anderson 1999; Ó Gráda 2008; Woods 1984). In pre-industrial England, prior to the onset of fertility decline, marital fertility did not differ much among occupational groups (Wrigley et al. 1997). Without fully considering the changing composition of the English family reconstitution sample over time, recent explorations of fertility differences in preindustrial England found that the higher social classes were likely to have larger families than other social classes; but by the early nineteenth century, social class differences in family size had diminished (Boberg-Fazlic et al. 2011; Clark and Cummins 2009; Clark and Hamilton 2006). The main sources used to study class differences in fertility during the fertility transition have also been sample populations for specific places in the mid–nineteenth century censuses or aggregate data from the 1911 Census Report on the Fertility of Marriage for England and Wales (Garrett et al. 2001; Haines 1979, 1992; Innes 1938; Stevenson 1920; Woods 1984; Woods and Smith 1983).

Using data from the published 1911 Census Report on the Fertility of Marriage (1917, 1923), Szreter’s (1996) comprehensive study of fertility in Britain downplayed the importance of social class as a determinant of differences in fertility, favoring instead communication communities. Recent work by Barnes and Guinnane (2012) has challenged these results, arguing that as much as two-thirds of the variation between couples in marital fertility was explained by social class. The debate that followed in the *Economic History Review* highlights the continued interest and complexity of the importance of social class and socioeconomic status in determining changes in family size (Barnes and Guinnane 2017; Szreter 2015). Although the authors disagreed on various points, their work has emphasized that geography and community differences within and between regions both have important roles in determining patterns of fertility, highlighting the need for further research using large-scale individual-level data to account for the interplay between geography and social class.

Work by Garrett et al. (2001) on individual-level data from 13 English and Welsh communities in 1891, 1901, and 1911 also suggested that place and class worked in tandem to produce patterns of fertility behavior: for example, the fertility of middle-class couples living in predominantly working class areas more closely resembled that of their lower-class neighbors than that of the middle class in general. Although this work was able to establish nuanced geographical and social patterns, it was limited to a small and disparate group of noncontiguous places. In addition, because they were an amalgam of both spatial and social factors, the dimensions of the spatio-social groups—referred to by Szreter (1996) as *communication communities*, and dubbed *environments* by Garrett et al. (2001)—remained unclear. It became evident that a finely grained analysis with much wider geographical coverage, identifying the occupational or social mix of relatively small spatial units, was essential to the identification of the forces determining variations and changes in fertility.

This work contributes to this debate by demonstrating how geographical patterns at a finer scale can enhance our understanding of historical fertility decline in England and Wales. This is done in two ways. On one hand, we focus on how the interplay between socioeconomic status and geographical context determined patterns of behavior and childbearing during the fertility transition. On the other hand, we shed new light on the effect of spatial mobility on individual fertility behavior.

## Data and Methods

### Integrated Census Microdata (I-CeM)

Our main data source is individual-level census data for England and Wales from 1851–1911 (except 1871, for which only very limited data are currently available for research purposes), provided via the Integrated Census Microdata (I-CeM) project (Higgs et al. 2013; Schürer and Higgs 2014).^1^ Each individual’s census record includes information on sex, age, marital status, occupation, place of residence, place of birth, and relationship to the household head. The latter variable makes it possible to link each married woman to her spouse and children if living in the same household. Our analysis is based on an enhanced version of the original I-CeM data in which household variables have been more precisely specified and individuals have been more accurately allocated to the Registration Sub-District (RSD) where they were recorded by the census.^2^ The population of England and Wales more than doubled over the 60-year period from 1851 to 1911. In the I-CeM database, the 1851 population was approximately 17.5 million in about 3.7 million households; by 1911, the population had increased to 36 million in almost 8 million households. The I-CeM database provides near-complete census coverage. However, a small number of original census pages have been lost or destroyed; in a few cases, full enumeration districts, parishes, or even whole RSDs are affected.^3^

## Measures

### Recent Marital Net Fertility

Because of the way census data were collected prior to 1911, our analysis of fertility differences has to rely on the number of a woman’s surviving children currently living in the same household with her rather than on the number of children ever born (although this latter measure was reported in 1911 because of the special questions on fertility-of-marriage asked in that census). We consider only children under age 5 because in the nineteenth century living away from parents increased after that age. Nevertheless, a small percentage of children aged 0–4 (5% to 10% in all census years) were not living with their parents, most likely because of orphanhood or illegitimacy, or because they were staying with relatives or friends during the census enumeration. Thus *own children within the household* is a measure of recent net fertility and has found common use in studies of fertility decline using historical individual-level census data (Dribe and Scalone 2014; Dribe et al. 2014; Hacker 2003, 2016). It is net because it does not take account of any mortality experienced by a woman’s children prior to the census enumeration (Reid et al. 2019). We acknowledge that differences in mortality between population subgroups—for example, by social class or place of residence—may have an impact on observed differences. For most social groups, fertility and mortality were positively correlated at an aggregate level, so the differences in fertility are slightly reduced when mortality is not taken into account. For example, higher social classes had both low fertility and low child mortality, whereas manual laborers and miners had large completed families and high levels of child mortality. Nevertheless, there are important exceptions: women married to textile workers, who had low fertility but relatively high infant and child mortality; and women married to agricultural laborers, who had high fertility and low infant and child mortality (Garrett and Reid 1994; Haines 1989; Woods and Smith 1983). Comparisons involving these groups must therefore be treated with more caution.

We calculate recent marital net fertility for each married woman aged 15–54 in each census, whose spouse was present in the same household.^4^ The husband’s presence was necessary because each woman’s socioeconomic status was derived from her husband’s occupational status: the great majority of married women did not report an occupation of their own. In 1851, 18% of married women gave an occupation, or were returned as economically active; but by 1911, this number had reduced to just 9%. Previous work has demonstrated that both changes in social attitudes and in the recording of female—especially married women’s—occupation played important role in declining labor force participation rates for women (Goose 2007; You 2014).

### Social Status

The census data provide extremely detailed information on male occupation, but we use the eight social classes first introduced by the Registrar General T. H. C. Stevenson to analyze fertility in his *Report* on the 1911 census enquiry into the *Fertility of Marriage* (Census of England and Wales 1911 1923)*.* The new classification evolved from previous occupational schemas, but in addition to the five graded classes, another three so-called industrial classes were given groups of their own (Szreter 1984). In broad terms, the eight classes are based on both social status and occupation: Class I, upper and middle classes (professional and managerial); Class II, skilled nonmanual workers (including farmers); Class III, skilled manual workers; Class IV, semiskilled manual workers; Class V, unskilled workers; Class VI, textile workers; Class VII, miners; and Class VIII, agricultural laborers. Stevenson singled out the last three categories specifically to analyze differences in fertility because they demonstrated unusual or extreme experiences within the working classes. Agricultural laborers were firmly part of the laboring classes, but unlike other laborers, they lived in healthy rural areas rather than in towns and cities, where mortality tended to be higher. Like agricultural laborers, miners had particularly high levels of fertility, but unlike agricultural laborers, they suffered high levels of child mortality. In contrast, textile workers were treated separately because their fertility was unusually low for the working classes (Szreter 1996).^5^ In this analysis, we apply this social class classification to all census years, which of course (perhaps erroneously) assumes that the occupations grouped together in 1911 were of a similar social status in 1851.

### Lifetime Migration

The measure of migration used in the analysis is calculated as a distance between a woman’s place of birth and where she was enumerated. Each individual born in England and Wales was asked to give their parish and county of birth in the census. Those born outside England or Wales were asked to give only country of birth. *Outside England and Wales* includes those born in Scotland and Ireland despite both countries being part of the United Kingdom at the time. This means that distance between place of birth and place of residence could be calculated for only those born in England and Wales and only for those who gave sufficient detail on their place of birth to allow this to be accurately identified. To produce lifetime migration measures from nineteenth century census data, the birthplace strings were standardized from 6.5 million plus unique strings to a smaller set of some 16,000 parish and county combinations (Schürer and Day 2019; Schürer et al. 2015). For each individual with a valid identifiable birthplace, a Euclidean distance (measured in kilometers (km)) was calculated between the centroids of the place of birth and the parish of enumeration.^6^ In our population of interest, the percentage of women born abroad ranged from 5% to 8%, and less than 1% of women had insufficient information to enable their place of birth to be identified (Day 2018b).

Table 1 demonstrates the age-standardized, mean net lifetime migration distances for women in each of the social classes from 1851 to 1911. As might be expected, women married to upper- and middle-class men had migrated the longest mean distances—roughly 65 km to 70 km—in each census year. It would appear that women married to men of lower social class lived much more local lives. The largest change in distance migrated shown in Table 1 is evident among miners’ wives, whose mean lifetime migration almost doubled between 1851 and 1881, from 21 to 38 km. This shift was primarily due to the growth of the mining industry, which was necessarily concentrated on the coalfields, necessitating an influx of new workers to such areas. By 1911, the mean lifetime migration of women married to agricultural laborers had increased to 29 km from a modest 15 km in the mid-nineteenth century.

**Table 1:** Age-standardized mean lifetime migration (in km) for married women aged 15–54 by social class of husband in England and Wales, 1851–1911

Husband’s Social Class	1851	1861	1881	1891	1901	1911
Upper and Middle Classes	66.35	69.66	70.77	69.01	66.09	63.44
Skilled Nonmanual	33.50	38.82	42.25	45.64	45.55	47.01
Skilled Manual	36.44	40.68	43.02	43.62	42.51	42.08
Semiskilled Manual	37.25	41.45	43.40	44.67	43.64	43.90
Unskilled	33.34	38.05	39.06	39.09	36.34	34.63
Textile Workers	19.04	21.14	21.42	23.87	24.48	24.42
Miners	20.62	25.82	38.43	37.57	34.42	35.77
Agricultural Laborers	14.83	19.10	17.62	19.40	22.67	28.80

*Source:* Calculated using Schürer and Higgs (2014) and Day (2018b).

### Registration Sub-Districts (RSDs)

The main geographical units used in our analysis are the RSDs. There were approximately 2,000 of these administrative units at each census year; they formed the basis of the civil registration system overseen by the Registrar General and were one of the reporting geographies for each decennial census between 1851 and 1911. RSDs varied considerably in size, ranging in area from fewer than 30 acres to well over 100,000 acres. They also varied in population size, from a few hundred people to 150,000 persons or more. Predominantly urban RSDs tended to be smaller in area but more populous. However, not all RSDs covered a uniformly urban or uniformly rural area: some contained part of a town as well as some of the surrounding area, and others that comprised mainly countryside included settlements of varying sizes. It is thus difficult to classify them as purely urban or purely rural environments. Over our study period, there was a considerable amount of redrawing of the RSD boundaries, especially between the 1891 and 1901 censuses when many—mostly urban—RSDs were merged to form larger units.

The constantly changing nature of RSDs means that we observe a different number of units at each census. Previous research on fertility decline in England and Wales has mostly used larger administrative units: counties (≈ 50 units) or registration districts (RD ≈ 600 units) as the main units of analysis (Glass 1938; Teitelbaum 1984; Woods 1987, 2000). RSDs provide a considerably more local context; therefore in this analysis, we use RSDs as a proxy for the local community to examine the impact of individual- and family-level characteristics on fertility behavior in the context of locality.

## Analysis

Our analytical strategy takes two approaches. First, we measure the relationship between marital net fertility and individual-level characteristics and investigate how it changed during the first few decades of the fertility transition. We estimate the same set of models for all available census years: 1851, 1861, 1881, 1891, 1901, and 1911. The analysis follows a fixed-effects modelling strategy where marital net fertility is the dependent variable, and the two main variables of interest are social class (*SC*) and distance from place of birth or lifetime migration (*LTM*). We also include a number of individual-level control variables (**X**_*t*_) in the model: age of woman, age difference between spouses, and husband’s household position (whether he was head of household):

1 $$ {y}_{ij}=\upalpha +{\upbeta}_1{SC}_{ij}+{\upbeta}_2{LTM}_{ij}+{\upbeta}_t{\mathbf{X}}_{tij}+{\upgamma}_j+{\upvarepsilon}_{ij}, $$

where *i* refers to a woman, *j* refers to her RSD of residence, and γ_*j*_ is the RSD unit fixed effect.^7^ We include the RSD-level fixed effects to control for structural differences and unobserved heterogeneity across geographical units, meaning that the identification of the models is based entirely on variations in marital net fertility within RSDs.^8^ The two variables, age of woman and age difference between spouses, control for age dependencies in fertility. Husband’s household position is a proxy for household resources, on the basis that a woman whose husband was not head of household would have been more likely to be young, very recently married, or suffering from financial hardship and limited access to resources, such as housing or childcare. In addition to the models that include all married women with spouse present, we estimate a separate set of models for each social class to investigate the extent to which differences in fertility by distance from place of birth can be explained by class-specific migration trajectories.^9^

Table 2 presents descriptive statistics for all the variables included in the analysis. The two main variables of interest are husband’s social class and distance from wife’s place of birth. More than 60% of all the women observed at each census were married to men in skilled (manual and nonmanual), semiskilled and unskilled occupations (Classes II–V), but the table also reveals considerable variation between the social classes across census years. As might be expected in an urbanizing and industrializing country, there was considerable decline in the absolute, and relative, numbers of women married to agricultural laborers between 1851 and 1911. Textile workers were the only class that did not change much over the period in absolute numbers, but they did experience a small decline in relative terms. Women married to upper- and middle-class men and to miners experienced the greatest growth. As expected from the long-standing tradition of neo-local marriage, the vast majority of husbands were head of their own household, and the percentage who were not decreased a little over time.

**Table 2:** Distribution of variables (%)

	1851	1861	1881	1891	1901	1911
Number of Children						
0	46.41	47.28	46.58	48.95	51.98	55.54
1	27.54	27.04	25.63	26.38	27.34	26.93
2	21.45	20.90	21.64	19.38	16.60	14.23
3	4.35	4.50	5.74	4.94	3.83	3.07
4	0.24	0.28	0.40	0.34	0.25	0.23
5+	0.01	0.01	0.01	0.01	0.01	0.01
Age Group of Wife						
15–19	0.66	0.83	0.85	0.60	0.43	0.28
20–24	9.53	10.21	10.54	9.52	8.66	6.67
25–29	17.16	16.91	17.87	17.53	17.60	15.96
30–34	18.50	17.97	17.92	18.28	18.89	19.25
35–39	16.95	16.72	16.47	17.02	17.44	18.48
40–44	15.05	15.26	14.74	14.77	14.94	15.90
45–49	12.17	12.37	11.92	12.35	12.32	13.18
50–54	9.98	9.73	9.68	9.93	9.72	10.28
Age Difference Between Spouses						
Wife older	24.78	23.85	22.97	22.47	21.98	22.45
Husband 0–2 years older	32.15	32.21	35.19	36.26	38.01	38.17
Husband 3–5 years older	20.27	20.20	20.73	20.82	21.09	21.11
Husband >6 years older	22.80	23.74	21.12	20.44	18.92	18.27
Husband’s Household Position						
Head of household	97.71	98.36	96.32	98.30	98.23	98.17
Other	2.29	1.64	3.68	1.70	1.77	1.83
Husband’s Social Class						
Upper and middle classes	4.74	4.88	7.31	7.90	8.96	10.16
Skilled nonmanual	17.09	16.17	16.03	16.11	15.96	16.04
Skilled manual	22.36	22.29	24.18	24.27	24.99	23.63
Semiskilled manual	12.22	12.69	15.36	15.76	16.72	17.16
Unskilled	13.12	14.13	16.53	17.05	16.45	15.67
Textile workers	7.02	6.09	4.41	4.07	3.61	3.34
Miners	4.18	4.88	5.69	6.31	6.85	8.05
Agricultural laborers	16.23	13.54	7.63	5.91	4.12	3.76
Unknown	3.03	5.33	2.87	2.61	2.34	2.18
Distance From Wife’s Place of Birth						
Less than 10 km	53.46	49.15	48.69	48.63	49.41	48.62
10–49 km	24.73	24.16	22.85	22.91	22.75	22.95
50+ km	16.33	19.18	22.00	23.15	23.17	23.54
Abroad	5.35	7.32	6.18	5.16	4.57	4.85
Unknown	0.13	0.19	0.28	0.14	0.10	0.05
Number of Women	2,223,976	2,541,617	3,235,007	3,507,452	4,111,065	4,659,742
Number of RSDs	2,176	2,189	2,175	2,110	2,060	2,009
Total Population	17,565,129	19,320,569	25,954,690	28,902,862	32,315,517	36,031,749

*Source*: Calculated using Schürer and Higgs (2014) and Day (2018b).

We note change in the distance that women migrated between birth and enumeration in the census. The percentage of women residing less than 10 km from their place of birth declined from 53% in 1851 to 49% in 1911. The main increase was in the category of long-distance migration (50+ km), which rose from 16% of women in 1851 to 24% in 1911. Thus, the absolute number of married women who had migrated 50+ km from their place of birth to their place of residence was more than three times higher in 1911 than in 1851.

Increases in age at marriage and reductions in adult male mortality mean that in the later censuses, our population of currently married women aged 15–54 included a larger proportion of older women: the percentage of 20- to 24-year-olds declined from approximately 10.5% in 1881 to only 6.7% in 1911. The age difference between spouses decreased over time; the percentage of women married to a man of similar age (husband 0–2 years older) increased from 32% in 1851 to 38% in 1911. This was at the expense of large age gaps (of more than six years) between spouses, probably because declining mortality meant that there were fewer couples composed of a widower and his younger second wife.

## Results

**Fig. 2:**
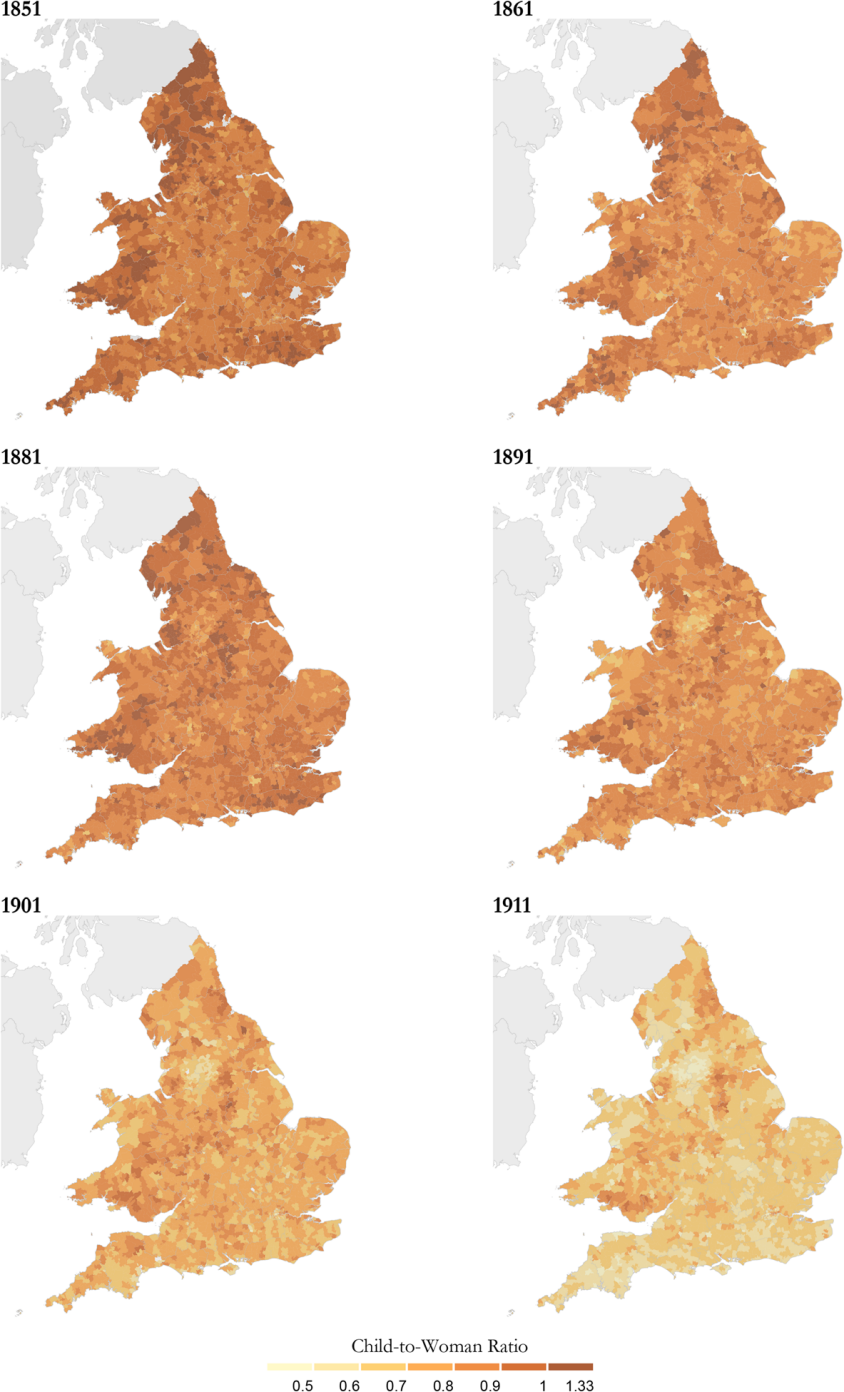
Child-woman ratios (children aged 0–4 per married-spouse-present woman aged 15–54) in RSDs, England and Wales, 1851–1911. *Base maps*: RSD boundaries for England and Wales. The RSD boundaries for England and Wales, 1851–1911, used for Fig. 2 were created by Dr. Joseph Day as part of the *Atlas of Victorian Fertility Decline*’ project (PI: A.M. Reid) with funding from the ESRC (ES/L015463/1) using Satchell et al. (2016), which is an enhanced version of Burton and Southall (2004), which itself is largely derived from Kain and Oliver, R. R. (2001). *Source:* Calculated using Schürer and Higgs (2014).

One of the indirect demographic measures most frequently employed when describing net fertility differences using census data is the *child-woman ratio* (CWR) (Scalone and Dribe 2017; Shryock and Siegel 1980), defined as the number of surviving children aged 0**–**4 per 1,000 married women aged 15–54. We used the CWR calculated from married-spouse-present women and their children, to explore spatial patterns at the RSD level. Figure 2 shows the changing levels of net fertility in England and Wales for all the available census years. During the first stages of fertility decline, the overall distribution of CWRs clearly shifted. The CWRs were less than 0.8 in most RSDs by 1911, whereas they were mainly higher than 0.8 or 0.9 before 1881. As expected, an early decline is visible in the textile districts of Lancashire and West Yorkshire. Mining centers stand out with high net fertility throughout the period, the main coalfields being located in South Wales, Durham, and along a spine running through Yorkshire, Derbyshire, and Nottinghamshire.^10^ The spatial fertility pattern is clearly underlined by the very distinct occupational geography of England and Wales, where certain industries came to dominate in particular regions. These regional patterns of occupational structure shaped local employment opportunities for men and women. In most textile areas, for instance, female labor force participation rates were high, with opportunities to remain at work after marriage or return to work after having had a child, and nuptiality and marital fertility rates were lower than in most working-class districts (Woods 1987). In addition to the textile areas, large urban centers, such as London, also demonstrate lower CWRs; however, these are not clearly visible on national scale maps such as those in Fig. 2.

### Social Class Differences in Fertility

Table 3 provides the CWR by social class over time. Most social classes experienced declines in net fertility after 1881 or 1891, but the upper and middle classes and textile workers had undoubtedly the earliest and fastest declines. Meanwhile, the women married to miners and unskilled workers experienced relatively slow declines, which started somewhat later in the period. These CWR patterns demonstrate considerable widening of relative class differences in the number of young children born, surviving, and still resident in the home during the early phases of fertility transition. For example, in the first three censuses, there was a difference of roughly 25% between the low CWR of the upper and middle classes and the high CWR of the miners, but this difference increased to 43% in 1891, 70% in 1901, and 86% in 1911. This is largely driven by the particularly rapid decrease in net fertility of women married to upper- and middle-class men.

**Table 3:** Child-woman ratios (children aged 0–4 per married-spouse-present woman aged 15–54) by social class of husband and spatial autocorrelation measures, England and Wales, 1851–1911

Husband’s Social Class	1851	1861	1881	1891	1901	1911
Child-Woman Ratios (CWR)						
Upper and middle classes	0.801	0.812	0.832	0.697	0.564	0.491
Skilled nonmanual	0.784	0.784	0.809	0.727	0.637	0.561
Skilled manual	0.857	0.847	0.923	0.841	0.747	0.657
Semiskilled manual	0.865	0.862	0.915	0.838	0.751	0.660
Unskilled	0.830	0.840	0.881	0.862	0.818	0.772
Textile workers	0.826	0.806	0.829	0.760	0.636	0.536
Miners	1.032	1.003	1.036	0.997	0.961	0.915
Agricultural laborers	0.931	0.901	0.925	0.890	0.828	0.748
Total	0.845	0.835	0.878	0.814	0.731	0.655
Moran’s *I* of CWRs						
Upper and middle classes	0.04	0.02	0.09	0.03	0.11	0.13
Skilled nonmanual	0.28	0.26	0.22	0.23	0.30	0.33
Skilled manual	0.27	0.23	0.23	0.18	0.21	0.29
Semiskilled manual	0.16	0.14	0.16	0.15	0.23	0.31
Unskilled	0.13	0.12	0.18	0.22	0.15	0.21
Total	0.52	0.48	0.51	0.50	0.53	0.60

*Source:* Calculated using Schürer and Higgs (2014) and Day (2018b).

We also investigated the extent of possible spatial clustering of CWRs by social class by deriving Moran’s *I* indices on the RSD-level class-specific measures (Table 3).^11^ We estimated Moran’s *I* only for the first five classes because these groups were present in most districts. As expected, these measures show relatively strong positive spatial autocorrelation. The upper and middle classes again stand out with the lowest Moran’s *I* values in each census year. This suggests that fertility levels, and possibly also patterns of fertility decline, among the higher social classes were largely unaffected by geography. Spatial fertility patterns among the other classes were, however, more strongly related to geography. These spatial differences also clearly emerge on maps of CWR by social class (see Figs. A2–A6 in the online appendix). The decline of CWR for skilled, semiskilled, and unskilled occupations is much more concentrated in textile areas, most likely shaped by local employment opportunities for women before marriage in those districts.

**Fig. 3:**
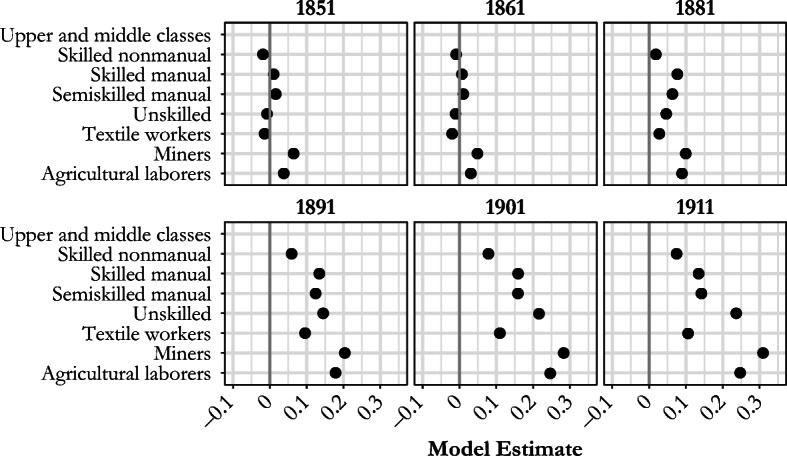
Model estimates for the relationship between marital net fertility (number of children aged 0–4) and the husband’s social class, England and Wales, 1851–1911. Models control for age of woman, age difference between spouses, household status, and wife’s distance from place of birth. Full model results in the online appendix (Table A2). *Source:* Calculated using Schürer and Higgs (2014) and Day (2018b).

Figure 3 presents the results of the fixed-effects models, which demonstrate that the differences in net fertility between the social classes were minimal during the first two decades of our observation period. With the onset of fertility decline, however, large differences emerged in net fertility, and these remained substantial even when individual-level demographic control variables were included in the model and when the identification reflected only marital net fertility differences within RSDs.^12^ Women married to upper- and middle-class men (the reference category) experienced the lowest net fertility from the 1881 census onward. The wives of miners and agricultural laborers had the highest net fertility and the slowest declines over the same period; the CWRs (see Table 3) also show that miners started to experience declining fertility considerably later than other social groups—not until the early twentieth century. Haines (1979) argued that the distinct demographic behavior of miners was mainly influenced by the nature of the employment opportunities available in their local areas, which meant that earnings for men peaked at young ages, young children had earning potential (especially in the mid-nineteenth century), and there was both an absence of female employment and a male-centered culture. The fact that mines were generally situated in largely rural areas also had implications for the mining communities: it made them socially and geographically isolated. Similar patterns have also been observed among miners in other countries on mainland Europe (Haines 1979; Wrigley 1961).

Unskilled workers also exhibited late onset of smaller family size, but they started from lower levels of fertility in the pre-transition period. Their slow fertility decline means that by 1911, women married to unskilled workers had joined miners’ and agricultural laborers’ wives in having the highest fertility. One explanation for this may be that the unskilled workers were experiencing high infant and early childhood mortality rates before the 1880s, but in the next few decades, their child survival improved more quickly than their fertility declined. It is clear that over time, the net fertility of the upper and middle classes became increasingly distinct from those of the lower orders. Women married to textile workers also diverged from the other manual classes, but their fertility decline was not as fast as that experienced by the upper and middle classes over the period studied. Overall, socioeconomic differences in net fertility widened during the first stages of the fertility transition (Skirbekk 2008). The results highlight that the fertility decline within the working classes was more occupation-specific than class-specific,^13^ making it important to separate textile workers from other manual laborers when analyzing changing fertility behavior in England and Wales.

One of the main limitations of using only the number of surviving children enumerated with mothers at the time of census to study social class differences in fertility is the potential influence of differential mortality experiences—in particular, the lack of a uniformly positive correlation between fertility and early age mortality. We wanted to ensure that the social class patterns revealed by our analysis of net fertility were not driven by differences in early age mortality, and the additional questions about fertility in the 1911 census allowed us to run a number of sensitivity tests to examine whether the observed social gradient in fertility holds even when we account for mortality. The enumerators of the 1911 census collected data from all married women on children ever born during their current marriage, children surviving, and marital duration. Unfortunately, the census does not provide information about when children were born or, if any died, their age at death (Census of England and Wales 1911 1917, 1923). However, using these data, we can estimate two additional models to compare the social gradient in fertility obtained with different measures of fertility. We used a more limited population of women who, in 1911, had been married for less than five years and for whom net achieved fertility over the duration of their marriage reflected recent net fertility. As before, we limited our analysis to women with husbands present on census night.

**Fig. 4:**
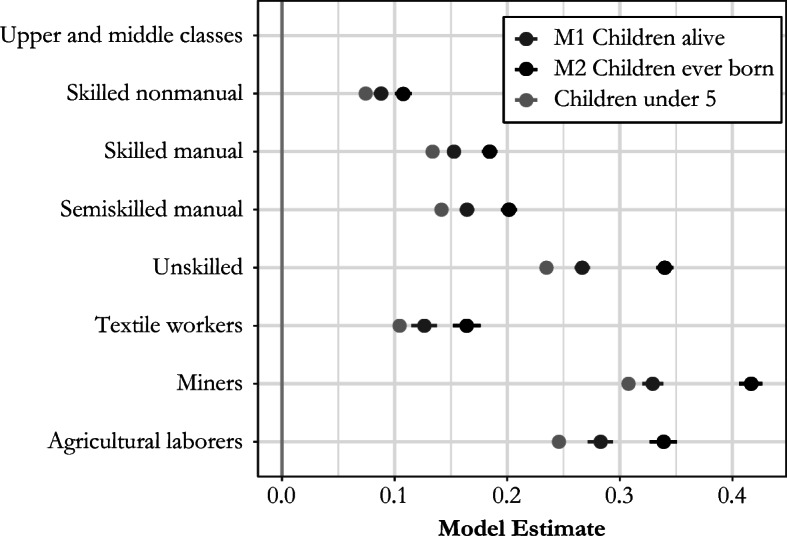
Model estimates for the relationship between the number of children ever born/children alive per couple and the social class of husband for women aged 15–54 who were married less than five years compared with the main model estimates (of children under 5) in England and Wales, 1911. Models control for age of woman, age difference between spouses, household status, and lifetime net migration. *Source:* Calculated using Schürer and Higgs (2014).

The first model (M1) uses net achieved fertility as the dependent variable, using only the number of children alive at the time of the 1911 census born to those women aged 15–54 who had been married for less than five years. The second model (M2) uses another measure of fertility for the same population of women: the total number of children ever born, which also includes the children a woman might have lost prior to the census enumeration. Figure 4 also shows the results of the main model (labelled *children under 5*). The difference between the main model and M1 is that the latter uses only recently married women, whereas the main model uses women of all marital durations. We would expect recently married women to have higher fertility in the last five years for two main reasons. First, they are younger and more fecund, which is controlled for by the inclusion of age in the models. Second, the interval from marriage to first birth tends to be smaller than the intervals between births because there is no postpartum or lactational infecundability, and this is the reason for the differences between the main model and M1 estimates in Fig. 4. Engagement in premarital intercourse also reduces the interval between marriage and first birth, and the larger gaps between the main model and M1 for agricultural laborers and unskilled workers may indicate a higher prevalence of premarital pregnancy among these classes.

Differences between M1 and M2 in Fig. 4 result purely from differential effects of mortality: because the upper and middle classes had the lowest risk of child mortality overall in 1911, using net fertility instead of total fertility dampens the social class differences in fertility, and this effect is strongest for groups with higher mortality, such as miners (Reid 1997). However, it is also clear that the overall social gradient and relative differences in fertility are very similar when using these different measures of fertility, although they were muted when net fertility is used. This supports our supposition that the social class differences we identify in marital net fertility also hold for marital fertility and are not distorted by differential mortality in different social groups.

### The Role of Migration in Fertility Decline

**Fig. 5:**
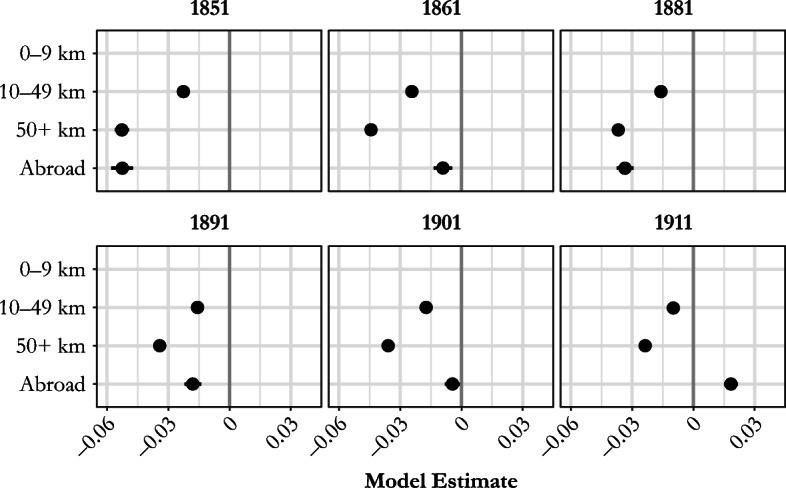
Model estimates for the relationship between marital net fertility (number of children aged 0–4) and wife’s distance from place of birth, England and Wales, 1851–1911. Models control for age of woman, age difference between spouses, household status, and husband’s social class. Full model results in the online appendix (Table A2). *Source:* Calculated using Schürer and Higgs (2014) and Day (2018b).

The net lifetime migration variable in Fig. 5 demonstrates interesting patterns. In all census years, the reference category—women residing within 10 km of their place of birth—had the highest net fertility. There is a clear gradient in the relationship between recent marital net fertility and the distance from place of birth: longer distances migrated were associated with lower net fertility. During the period between 1851 and 1901, the gradient and differences were fairly stable but diminished somewhat by 1911. Overall, our results do not indicate a distinct change in the role of migration during the first decades of fertility transition, after 1881; instead, we find that the differences evident before that date remained largely intact across the next three decades.^14^

**Fig. 6:**
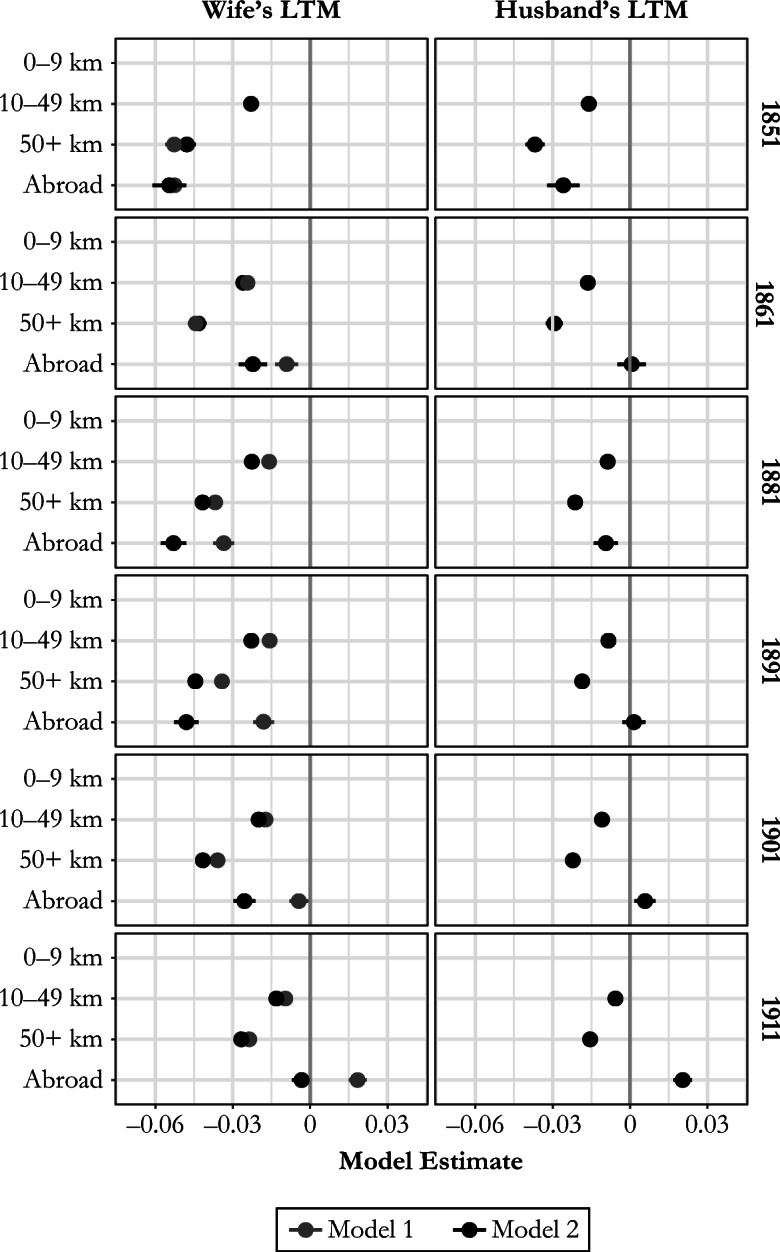
Model estimates for the relationship between marital net fertility (number of children aged 0–4) and both wife’s and husband’s distance from place of birth (lifetime migration = LTM), in kilometers (km), England and Wales, 1851–1911. Model 1 includes only wife’s distance from place of birth. Model 2 includes both wife’s and husband’s distance from place of birth. Both models control for age of woman, age difference between spouses, household status, and husband’s social class. *Source:* Calculated using Schürer and Higgs (2014) and Day (2018b).

We also tested whether a couple’s fertility behavior might have been independently influenced by the husband’s lifetime migration. The results of these models are presented in Fig. 6. For those born in England and Wales, inclusion of the husband’s migration makes little difference to the effects of the wife’s migration, indicating that the latter has a largely independent effect on fertility. The effects of husbands’ lifetime migration on fertility shows a similar pattern to that of their wives, but the differences in net fertility are much smaller for husbands’ migration, confirming our expectation that women’s own migration trajectories were much more influential than those of their husbands in determining net marital fertility. For those born overseas, however, the effects are somewhat different. Here, the effect of husbands’ migration seems to confound that of wives’: the effects for wives increase after husbands’ migration is controlled. We suspect that different countries of origin and migration patterns over time make this a product of a variety of different experiences, and these will be explored in subsequent research.

**Fig. 7:**
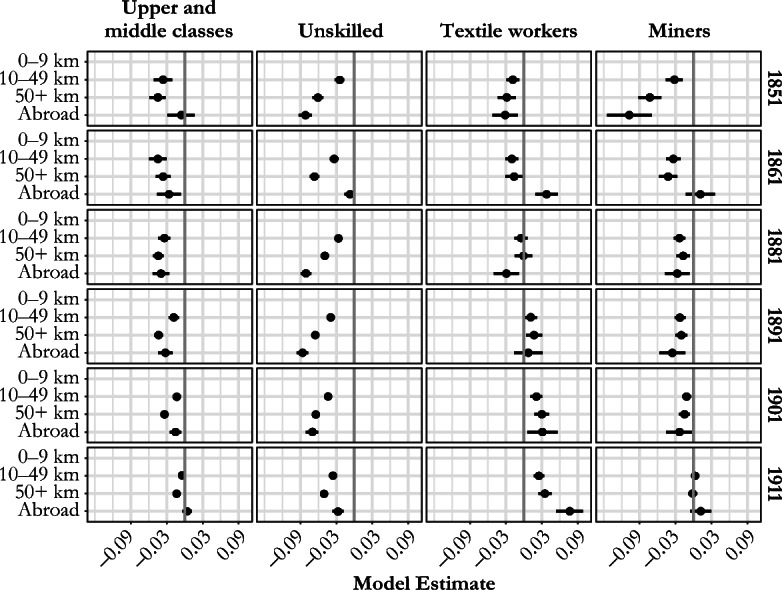
Model estimates for the relationship between marital net fertility (number of children aged 0–4) and wife’s lifetime migration distance, by husband’s social class, England and Wales, 1851–1911. Models control for age of woman, age difference between spouses, and husband’s status within the household. *Source:* Calculated using Schürer and Higgs (2014) and Day (2018b).

To explore whether the differences in net fertility were more responsive to the length of lifetime migration among some social classes than among others, we estimated a separate set of models for each social class in every census year. The national pattern in the fertility-migration relationship is largely guided by differences in the first five social classes; they demonstrate the same gradient across all census years. The exceptions are the three industrial classes (miners, agricultural laborers, and textile workers), who also exhibited the shortest mean net lifetime migration distances across all census years (see Table 1). The new sets of model estimates for the women married to textile workers, miners, unskilled workers, and the upper and middle classes are shown in Fig. 7. (Model estimates for all class-distance combinations are shown in Fig. A9, online appendix.) A longer distance migrated from place of birth to place of residence at the time of enumeration was associated with lower fertility for the unskilled workers, with a clear gradient from less than 10 km, through 10–49 km to 50+ km. This pattern also holds for skilled and semiskilled workers (Fig. A9). The differences in fertility by distance from place of birth were somewhat smaller among upper- and middle-class women. Locally born miners’ wives also had higher fertility than those who had migrated from elsewhere in England and Wales, although the effects are small with no clear gradient with increasing distance. In contrast, women married to textile workers were the only social class for whom being born locally was associated with lower fertility: from 1891 onward, textile workers’ wives born 50+ km from their place of residence had significantly higher fertility than their locally born neighbors.

## Discussion

We used individual-level historical census data for England and Wales to explore the interplay among social class, migration, geography, and fertility to gain a greater understanding of differences in net fertility during the second half of the nineteenth and the early twentieth centuries. The main limitations of using census data to study individual-level fertility are that the analysis is based on cross-sectional data at 10-year intervals and that mortality information is lacking. Nevertheless, in our sensitivity tests and also in previous work on estimating social class differences in fertility using the own-children method where mortality rates are adjusted for, we obtained very similar patterns of fertility (Reid et al. 2019).

Our analyses confirm a clear pattern of widening social class differences in recent net fertility during the first decades of the fertility transition. These findings provide strong support for the argument that belonging to a certain social group was an important determinant of fertility behavior and of the timing of the onset of the reduction in family size within marriage in England and Wales. We find that class differences in recent net fertility were very narrow prior to the transition. However, women married to upper- and middle-class men and to textile workers initiated the move toward lower fertility, showing considerably lower net fertility than other social classes during the early phases of transition. On the other hand, wives of miners and agricultural laborers had the highest net fertility and did not exhibit any signs of fertility limitation until the early twentieth century.

It is encouraging that the results of this study are consistent with a plethora of previous work on class differences during fertility decline in England and Wales (Anderson 1999; Garrett et al. 2001; Haines 1992; Szreter 1996; Woods 1984). Furthermore, extended work on the Swedish fertility transition has also highlighted the role of the upper and middle classes in leading the way in reducing family size in the late nineteenth century (Dribe and Scalone 2014; Klüsener et al. 2019). Similarly, international comparisons have found exactly the same patterns of widening socioeconomic differences in fertility for other countries in North America and Europe (Dribe et al. 2014; Haines 1992). In general, these studies and our current results provide support for universal patterns of widening social class differences in fertility across different populations and similarities in how these evolved over the early phases of fertility decline.

The present study represents one of the first attempts to explore the relationship between individual migration patterns and fertility during the fertility transition in England and Wales. In contrast to the findings by Klüsener et al. (2019) for Sweden, our results reveal that longer distance net lifetime migration—measured using distance migrated from place of birth to place of residence—was associated with lower marital net fertility in England and Wales. We also find that this effect was relatively constant throughout the whole period, without a distinct change in the pattern at the onset of fertility decline. Therefore, it is difficult to suggest that long-distance migrants were the early adopters of family limitation with better access to information and new social attitudes toward fertility behavior. It is more likely that life course migration patterns determined observed fertility differences through the postponement of marriage and starting a family. However, it is important to consider that while major demographic changes were occurring in society, the meaning of distance was also transforming. Not only did the rapidly developing transport and communication networks transform the significance of short and long distance migration, thus allowing people to travel longer distances in shorter times, but the increasing numbers of women and their husbands who had undertaken moves from their place of birth to a new place of residence meant that information flows and knowledge fields must have been much more evenly distributed across the population by the end of our study period than they were at the beginning.^15^ As a result, new behavior could be transmitted increasingly swiftly across space and all levels of society.

In contrast to every other social class, however, textile workers’ wives who were born locally (less than 10 km from place of residence) exhibited increasingly lower fertility than those born farther away, and this pattern emerged at the onset of fertility decline. Using 1881 census data, Day (2015) suggested that in the latter part of the nineteenth century, migration into some textile towns was far less important than it was in earlier periods of growth. It seems that those few who did migrate from elsewhere were much less likely to have lower fertility than the local women who married textile workers. Possible explanations might be that women from farther away did not grow up where they could earn a living in the textile mills before marriage, or it might have been more difficult for nonnatives to obtain work in the mills due to discrimination, lack of contacts, or lack of skills. Overall, this suggests that the economic and social contextual factors of textile areas were important in shaping local fertility behavior over and above the life course migration patterns that might influence longer-distance migrants to postpone marriage and childbearing.

The early adoption of new reproductive behavior by upper- and middle-class couples and also by women married to textile workers is a strong indication that fertility transition in England and Wales was not dependent on just the process of innovation, initially accessible only to certain members of the upper classes of society. Banks’ (1981) argument of *direct diffusion*—the working classes copying the family limitation practices used by higher social classes—hardly seems convincing in this context. This is particularly the case when the motivations driving childbearing and child-rearing practices, which were shaped by local and class-specific experiences, were also dramatically changing over the second half of the nineteenth century (Pooley 2013). The distinct early fertility declines among upper- and middle-class women and the wives of textile workers compared with the wives of other manual workers is in line with the idea of *multiple fertility transitions* introduced by Szreter (1996). Rather than a simple socially graded single process, fertility decline in England and Wales occurred in different ways across different communities, and where spatial patterns of fertility transition were magnified by distinct residential patterns or occupational concentration of certain social classes in different parts of the country.

## Supplementary Information

ESM 1 (PDF 11.8 mb)

## Data Availability

Integrated Census Microdata (I-CeM) is publicly available from UK Data Archive. GIS datasets are available from UK Data Archive and the Cambridge Group for the History of Population and Social Structure.

## Footnotes

1 Schürer et al. (2018) and Reid et al. (2019) provide more information about the I-CeM database and in particular about how individual-level census data can be used to examine fertility.

2 The version of the I-CeM data used here has been enhanced as part of the ESRC-funded An Atlas of Victorian Fertility Decline project (ES/L015463/1) at the Cambridge Group for the History of Population and Social Structure, Department of Geography, University of Cambridge. For further details, see http://www.geog.cam.ac.uk/research/projects/victorianfertilitydecline/.

3 For 1851, data are missing for 14 RSDs with the combined population of 81,278 (0.5% of the total population of England and Wales); for 1861, 5 RSDs with the combined population of 95,148 (0.5% of the total population); and for 1901, 4 RSDs with population total of 46,754 (0.1% of the total population).

4 A table with information about the census population and our final study population is included in the online appendix (see Table A1).

5 In addition to these eight social classes, we also include a separate Unknown category for certain cases where the detailed information on occupation was missing or uncertain. This last group largely comprises those who had no recorded occupation, were living on their own means, were retired, or whose census entry was illegible.

6 Individuals matched to more than one parish (because, e.g., there were multiple parishes with the same name) were weighted between those parishes using the relative populations of each parish in the year of their birth. Overall, approximately 95% of the population in the I-CeM database were matched to a place of birth that was as precise as possible, with each individual having an estimate of a minimum (matched only to the nearest possible parish), average (population-weighted average of all possible parishes), and maximum (matched to the furthest of the possible parishes) distance migrated. We report average distance in all the calculations, but results using the minimum distance are very similar.

7 As the independent variable is based on count data, we also ran a number of sensitivity tests with a Poisson specification. Our model outcomes were the same in both modelling strategies. For results, see Table A3 in the online appendix.

8 To test whether our model results can be interpreted as changes in fertility behavior and not as the direct outcome of changes in the RSD geography, we ran a sensitivity analysis using a consistent geography across all census years. This was based on the 588 amalgamated registration districts used by Hinde and Harris (2019) to study mortality decline in the same period. The results are presented in Fig. A1 in the online appendix. It is reassuring that using a constant geography confirmed all our main findings and that the estimates for social class differences in net fertility were similar to the results from the main fixed-effects models (which used a changing number of RSDs across the census years).

9 All the data transformations and fixed-effects modelling are carried out in R version 3.4.2 (R Core Team 2017) using the *pglm* or *plm* packages (Croissant 2017; Croissant and Millo 2008).

10 As shown in Fig. 2, in 1911, the RSDs with lower fertility (lighter color) just to the northeast of Wales’ northern coast line are the textile districts. The RSDs with higher fertility (darker color) in South Wales, on England’s northeast coast and to the east and south of the textile areas, are mining areas.

11 The spatial weight matrices define neighbors by contiguity with first-order queen definition of adjacency. RSDs are neighbors when they share at least one common part of their border, and each neighbor is given equal weight within a matrix.

12 Sensitivity checks with ordinary least squares (OLS) models without fixed-effects specification (see Fig. A7 in the online appendix) demonstrate that a considerable part of the differences in net fertility among the lower social classes is explained by unobserved spatial heterogeneity measured at RSD level. However, the model estimates for the higher social classes are relatively similar regardless of whether fixed-effects models were used. These patterns prevail at every census year.

13 The Registrar General’s classification of social classes is rather misleading because in the end, the abnormal groups that did not fit the class gradient were separated to the three industrial classes.

14 Additional sensitivity checks, implementing models without any fixed-effects specification (see Fig. A8 in the online appendix), show the importance of spatial heterogeneity in net fertility estimates by distance from place of birth. However, over time, less of the variation in net fertility is explained by the structural differences across RSDs.

15 This can also be seen through marriage patterns. For example, Perry (1969) suggested that the level of exogamous marriages and the distance recorded between marriage partners increased significantly from the 1880s for working class marriage partners in rural Dorset. A similar transition can be seen in the urbanizing seaside resort of Southend-on-Sea (Schürer 1982).

## References

[CR1] Anderson M (1999). Highly restricted fertility: Very small families in the British fertility decline. Population Studies.

[CR2] Banks JA (1981). Victorian values: Secularism and the size of families.

[CR3] Barnes G, Guinnane TW (2012). Social class and the fertility transition: A critical comment on the statistical results reported in Simon Szreter’s *Fertility, class and gender in Britain, 1860–1940*. Economic History Review.

[CR4] Barnes G, Guinnane TW (2017). Rejoinder to Szreter. Economic History Review.

[CR5] Becker G (1981). A treatise on the family.

[CR6] Boberg-Fazlic N, Sharp P, Weisdorf J (2011). Survival of the richest? Social status, fertility and social mobility in England 1541–1824. European Review of Economic History.

[CR7] Burton, N., & Southall, H. R. (2004). *GIS of the ancient parishes of England and Wales, 1500–1850* [Data collection]. Colchester: UK Data Service. 10.5255/UKDA-SN-4828-1

[CR8] Carlsson G (1966). The decline of fertility: Innovation or adjustment process. Population Studies.

[CR9] Census of England and Wales 1911. (1917). *Vol. XIII, Fertility of Marriage, Part I*. London, UK: His Majesty’s Stationery Office.

[CR10] Census of England and Wales 1911. (1923). *Vol. XIII, Fertility of Marriage, Part II*. London, UK: His Majesty’s Stationery Office.

[CR11] Clark, G., & Cummins, N. (2009). Urbanization, mortality, and fertility in Malthusian England. *American Economic Review: Papers & Proceedings, 99,* 242–247. 10.1257/aer.99.2.242 29505219

[CR12] Clark G, Hamilton G (2006). Survival of the richest: The Malthusian mechanism in pre-industrial England. Journal of Economic History.

[CR13] Cleland J, Wilson C (1987). Demand theories of the fertility transition: An iconoclastic view. Population Studies.

[CR14] Coale AJ, Watkins SC (1986). The decline of fertility in Europe.

[CR15] Core Team R (2017). R: A language and environment for statistical computing.

[CR16] Creighton M, Matthys C, Quaranta L (2012). Migrants and the diffusion of low marital fertility in Belgium. Journal of Interdisciplinary History.

[CR17] Croissant, Y. (2017). *pglm: Panel generalized linear models (R package version 0.2-1)* [Data set]. Retrieved from https://CRAN.R-project.org/package=pglm

[CR18] Croissant Y, Millo G (2008). Panel data econometrics in R: The plm package. Journal of Statistical Software.

[CR19] Day J (2015). *Leaving home and migrating in nineteenth-century England and Wales: Evidence from the 1881 census enumerators’ books* (Doctoral thesis).

[CR20] Day, J. (2018a). Leaving home in 19th century England and Wales: A spatial analysis. *Demographic Research, 39,* 95–135. 10.4054/DemRes.2018.39.4

[CR21] Day J (2018). *Lifetime migration of England and Wales, 1851–1911* [Unpublished data set].

[CR22] Dribe M (2009). Demand and supply factors in the fertility transition: A county-level analysis of age-specific marital fertility in Sweden, 1880–1930. European Review of Economic History.

[CR23] Dribe M, Hacker JD, Scalone F (2014). The impact of socio-economic status on net fertility during the historical fertility decline: A comparative analysis of Canada, Iceland, Sweden, Norway, and the USA. Population Studies.

[CR24] Dribe, M., & Scalone, F. (2014). Social class and net fertility before, during, and after the demographic transition: A micro-level analysis of Sweden 1880–1970. *Demographic Research, 30,* 429–464. 10.4054/DemRes.2014.30.15

[CR25] Easterlin RA (1975). An economic framework for fertility analysis. Studies in Family Planning.

[CR26] Easterlin RA, Crimmins EM (1985). The fertility revolution: A supply-demand analysis.

[CR27] Galor O, Weil DN (2000). Population, technology, and growth: From Malthusian stagnation to the demographic transition and beyond. American Economic Review.

[CR28] Garrett EM, Reid A (1994). Satanic mills, pleasant lands: Spatial variation in women’s work, fertility and infant mortality as viewed from the 1911 census. Historical Research.

[CR29] Garrett E, Reid A, Schürer K, Szreter S (2001). Changing family size in England and Wales: Place, class and demography, 1891–1911.

[CR30] Glass DV, Hogben L (1938). Changes in fertility in England and Wales, 1851*–*1931. Political arithmetic.

[CR31] Goldstein JR, Klüsener S (2014). Spatial analysis of the causes of fertility decline in Prussia. Population and Development Review.

[CR32] Goose N (2007). Women’s work in industrial England: Regional and local perspectives.

[CR33] Gráda Ó, C. (2008). Economic status, religion, and demography in an Ulster town in the early twentieth century. History of the Family.

[CR34] Gregory IN, Henneberg JM (2010). The railways, urbanization, and local demography in England and Wales, 1825–1911. Social Science History.

[CR35] Hacker JD (2003). Rethinking the “early” decline of marital fertility in the United States. Demography.

[CR36] Hacker JD (2016). Ready, willing, and able? Impediments to the onset of marital fertility decline in the United States. Demography.

[CR37] Haines MR (1979). Fertility and occupation: Population patterns in industrialization.

[CR38] Haines MR (1989). Social class differentials during fertility decline: England and Wales revisited. Population Studies.

[CR39] Haines MR, Gillis JR, Tilly LA, Levine D (1992). Occupation and social class during fertility decline: Historical perspectives. *The European experience of declining fertilit*y*, 1850–1970: The quiet revolution*.

[CR40] Higgs EJ, Jones C, Schürer K, Wilkinson A (2013). Integrated census microdata (I-CeM) guide.

[CR41] Hinde A, Harris B (2019). Mortality decline by cause in urban and rural England and Wales, 1851–1910. History of the Family.

[CR42] Human Mortality Database. (2019). *The Human Mortality Database* [Data set]. Berkeley, CA (USA), and Rostock, Germany: University of California, Berkeley (USA), and Max Planck Institute for Demographic Research (Germany). Available from http://www.mortality.org/

[CR43] Innes JW (1938). Class fertility trends in England and Wales, 1876–1934.

[CR44] Kain, R. J. P., & Oliver, R. R. (2001). *Historic parishes of England and Wales: An electronic map of boundaries before 1850 with a gazetteer and metadata* [Data collection]. Colchester: UK Data Service. 10.5255/UKDA-SN-4348-1

[CR45] Klüsener S, Dribe M, Scalone F (2019). Spatial and social distance at the onset of the fertility transition: Sweden, 1880–1900. Demography.

[CR46] Kulu H (2005). Migration and fertility: Competing hypotheses re-examined. European Journal of Population/Revue européenne de Démographie.

[CR47] Mason KO (1997). Explaining fertility transitions. Demography.

[CR48] Morse, D. (1987). *The fertility decline in Scotland* (Unpublished doctoral dissertation). Edinburgh, Scotland: University of Edinburgh.

[CR49] Office for National Statistics. (2011). *Age at marriage and previous marital status 2011* [Data set]. Newport, South Wales: Office of National Statistics.

[CR50] Perry PJ (1969). Working-class isolation and mobility in rural Dorset, 1837–1936: A study of marriage distances. Transactions of the Institute of British Geographers.

[CR51] Pooley S (2013). Parenthood, child-rearing and fertility in England, 1850–1914. History of the Family.

[CR52] Reid, A. (1997). Locality or class? Spatial and social differentials in infant and child mortality in England and Wales, 1895–1911*.* In C. A. Corsini & P. P. Viazzo (Eds.), *The decline of infant and child mortality: The European experience: 1750*–*1990* (pp. 129–154). Hague, the Netherlands: Martinus Nijhoff Publishers.

[CR53] Reid AM, Garrett E, Szreter S, Fariñas DR, Oris M (2016). Residential mobility and child mortality in early twentieth century Belfast. New approaches to death in cities during the health transition.

[CR54] Reid, A., Jaadla, H., Garrett, E., & Schürer, K. (2019). Adapting the Own Children Method to allow comparison of fertility between populations with different marriage regimes. *Population Studies, 74*, 197–218. 10.1080/00324728.2019.1630563 31354068

[CR55] Satchell, A. E. M., Kitson, P. K., Newton, G. H., Shaw-Taylor, L., & Wrigley, E. A. (2016). *1851 England and Wales census parishes, townships and places* [Data collection]. Colchester: UK Data Archive. 10.5255/UKDA-SN-852232

[CR56] Scalone F, Dribe M (2017). Testing child-woman ratios and the own-children method on the 1900 Sweden census: Examples of indirect fertility estimates by socioeconomic status in a historical population. Historical Methods: A Journal of Quantitative and Interdisciplinary History.

[CR57] Schumacher, R., Matthijs, K., & Moreels, S. (2013). Migration and reproduction in an urbanizing context. Family life courses in 19th century Antwerp and Geneva. *Revue Quetelet/Quetelet Journal, 1*(1), 51–72.

[CR58] Schürer K (1982). The analysis of nineteenth-century marriage registers.

[CR59] Schürer K, van Poppel F, Oris M, Lee J (2003). Leaving home in England and Wales 1850*–*1920. The road to independence: Leaving home in eastern and western societies, 16th–20th centuries.

[CR60] Schürer K, Day J (2019). Migration to London and the development of the north-south divide, 1851–1911. Social History.

[CR61] Schürer K, Garrett E, Jaadla H, Reid AM (2018). Household and family structure in England and Wales (1851–1911): Continuities and change. Continuity and Change.

[CR62] Schürer, K., & Higgs, E. (2014). *Integrated Census Microdata (I-CeM), 1851–1911* [Data collection]. Colchester: UK Data Service. 10.5255/UKDA-SN-7481-1

[CR63] Schürer K, Penkova T, Shi Y (2015). Standardising and coding birthplace strings and occupational titles in the British censuses of 1851 to 1911. Historical Methods: A Journal of Quantitative and Interdisciplinary History.

[CR64] Shryock, H., Siegel, J., & Associates. (1980). *The methods and materials of demography* (Vol. 2). Washington, DC: U.S. Department of Commerce, Census Bureau.

[CR65] Skirbekk, V. (2008). Fertility trends by social status. *Demographic Research, 18,* 145–180. 10.4054/DemRes.2008.18.5

[CR66] Stevenson THC (1920). The fertility of various social classes in England and Wales from the middle of the nineteenth century to 1911. Journal of the Royal Statistical Society.

[CR67] Szreter S (1984). The genesis of the registrar-general’s social classification of occupation. British Journal of Sociology.

[CR68] Szreter S (1996). Fertility, class and gender in Britain, 1860–1940.

[CR69] Szreter S (2015). Fertility, social class, gender, and the professional model: Statistical explanation and historical significance. Economic History Review.

[CR70] Teitelbaum MS (1984). The British fertility decline: Demographic transition in the crucible of the Industrial Revolution.

[CR71] Wall R (1987). Leaving home and the process of household formation in pre-industrial England. Continuity and Change.

[CR72] Woods R (1979). Population analysis in geography.

[CR73] Woods, R. (1984). Social class variations in the decline of marital fertility in late nineteenth-century London. *Geografiska Annaler: Series B, Human Geography, 66,* 29–38.

[CR74] Woods R (1987). Approaches to the fertility transition in Victorian England. Population Studies.

[CR75] Woods R (2000). The demography of Victorian England and Wales.

[CR76] Woods R, Smith CW (1983). The decline of marital fertility in the late nineteenth century: The case of England and Wales. Population Studies.

[CR77] Wrigley EA (1961). Industrial growth and population change: A regional study of the coalfield areas of north-west Europe in the later 19th century.

[CR78] Wrigley EA, Davies RS, Oeppen JE, Schofield RS (1997). English population history from family reconstitution 1580–1837.

[CR79] You, X. (2014). *Women’s employment in England and Wales, 1851–1911* (Doctoral thesis). Cambridge, UK: Department of History, University of Cambridge.

