# Supplementary material for: Revisiting the Fertility Transition in England and Wales: The Role of Social Class and Migration
Source: Demography. 2020 Jul 1;57(4):1543–69. doi: 10.1007/s13524-020-00895-3 (PMC7441055; doi:10.1007/s13524-020-00895-3)
Supplement: Supplementary file 1 — (PDF 11.8 mb) [file 13524_2020_895_MOESM1_ESM.pdf]

Online Appendix

Fig. A1 Model estimates from fixed-effects models using consistent or changing administrative geography for the relationship between marital net fertility (number of children aged 0–4) and husband's social class, England and Wales, 1851–1911.

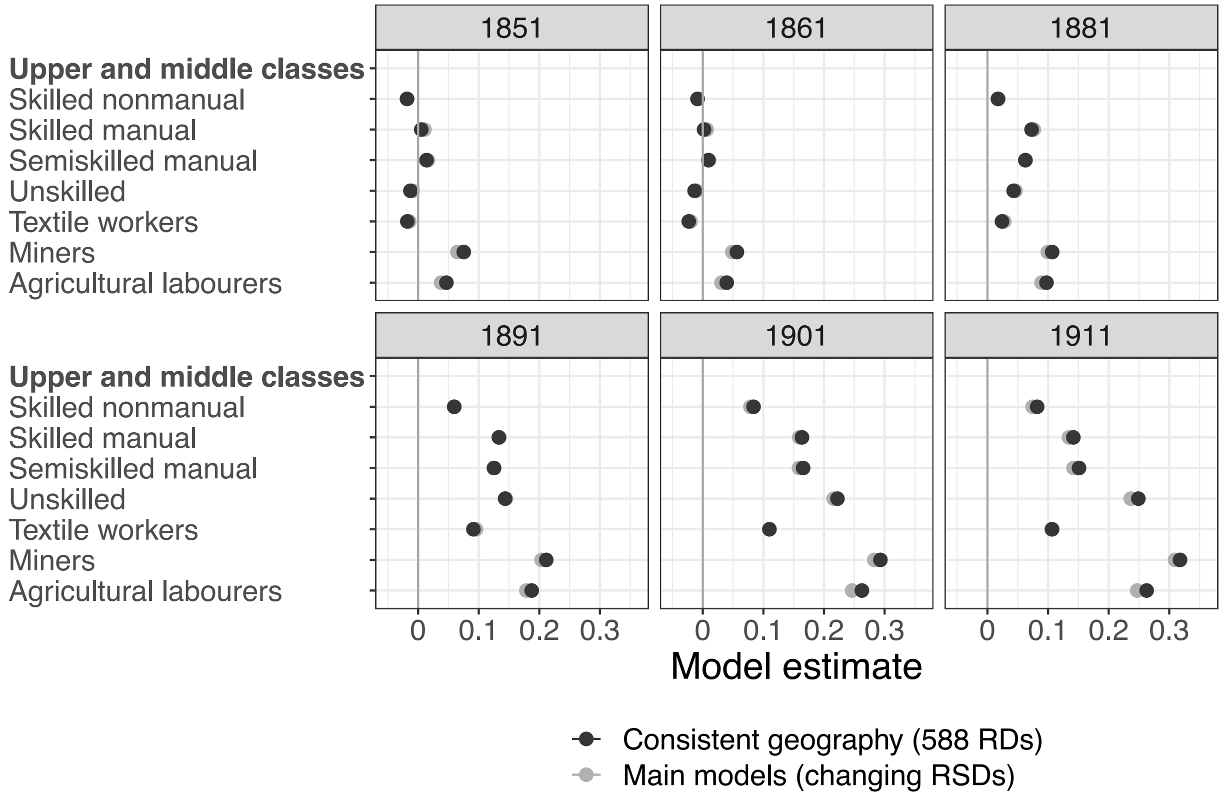

Note: *Models control for age of woman, age difference between spouses, household status, and husband's social class based on estimation on registration districts (RDs) or registration sub-districts (RSDs).*

Source: Calculated using Schürer and Higgs (2014) and Day (2018b).

Fig. A2 Child-woman ratios for upper and middle classes (children aged 0–4 per married spouse present women aged 15–54) in registration sub-districts, England and Wales, 1851–1911

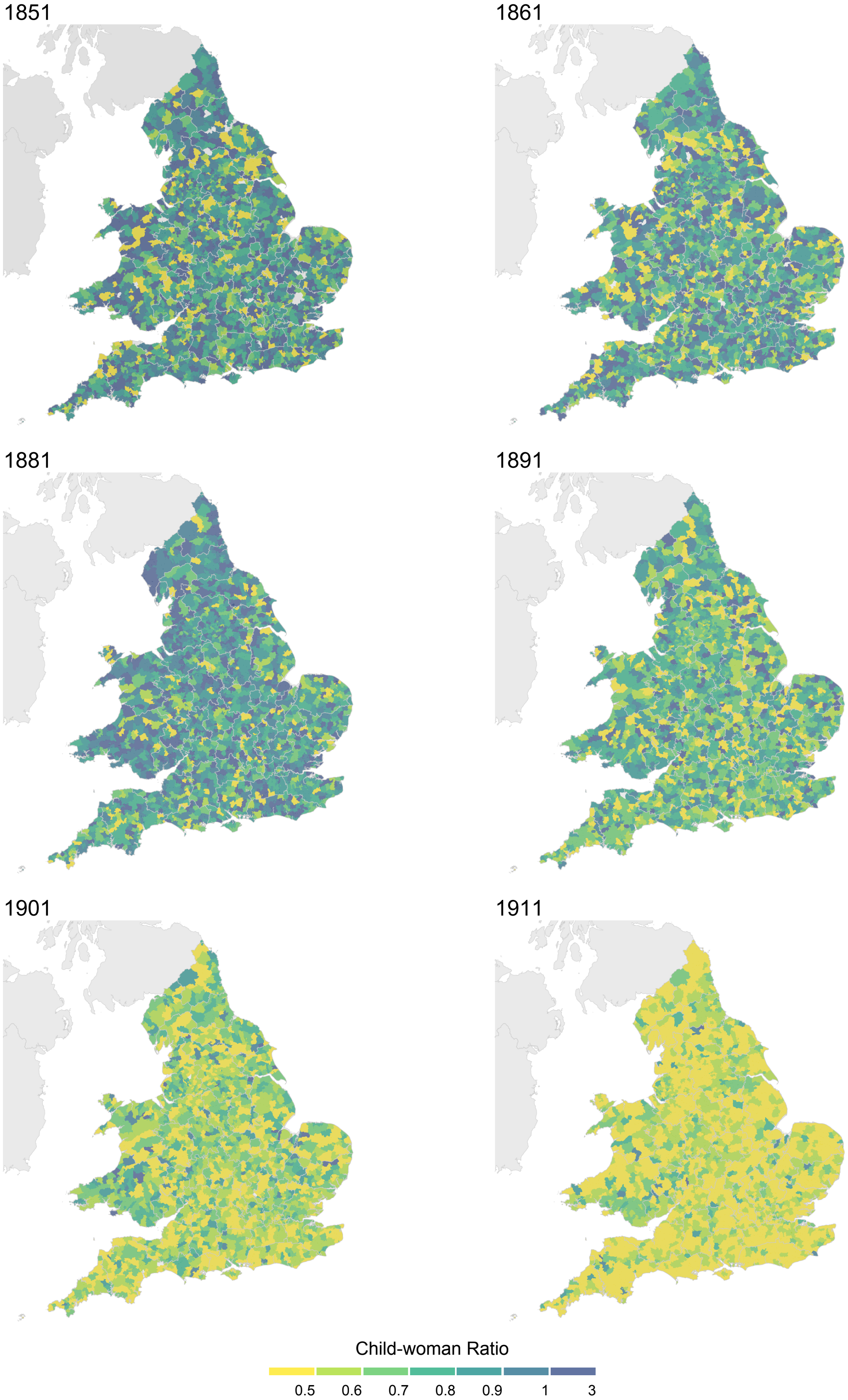

Source: Schürer and Higgs (2014)
Base maps: Registration sub-district boundaries for England and Wales

Fig. A3 Child-woman ratios for skilled nonmanual workers (children aged 0–4 per married spouse present women aged 15–54) in registration sub-districts, England and Wales, 1851–1911

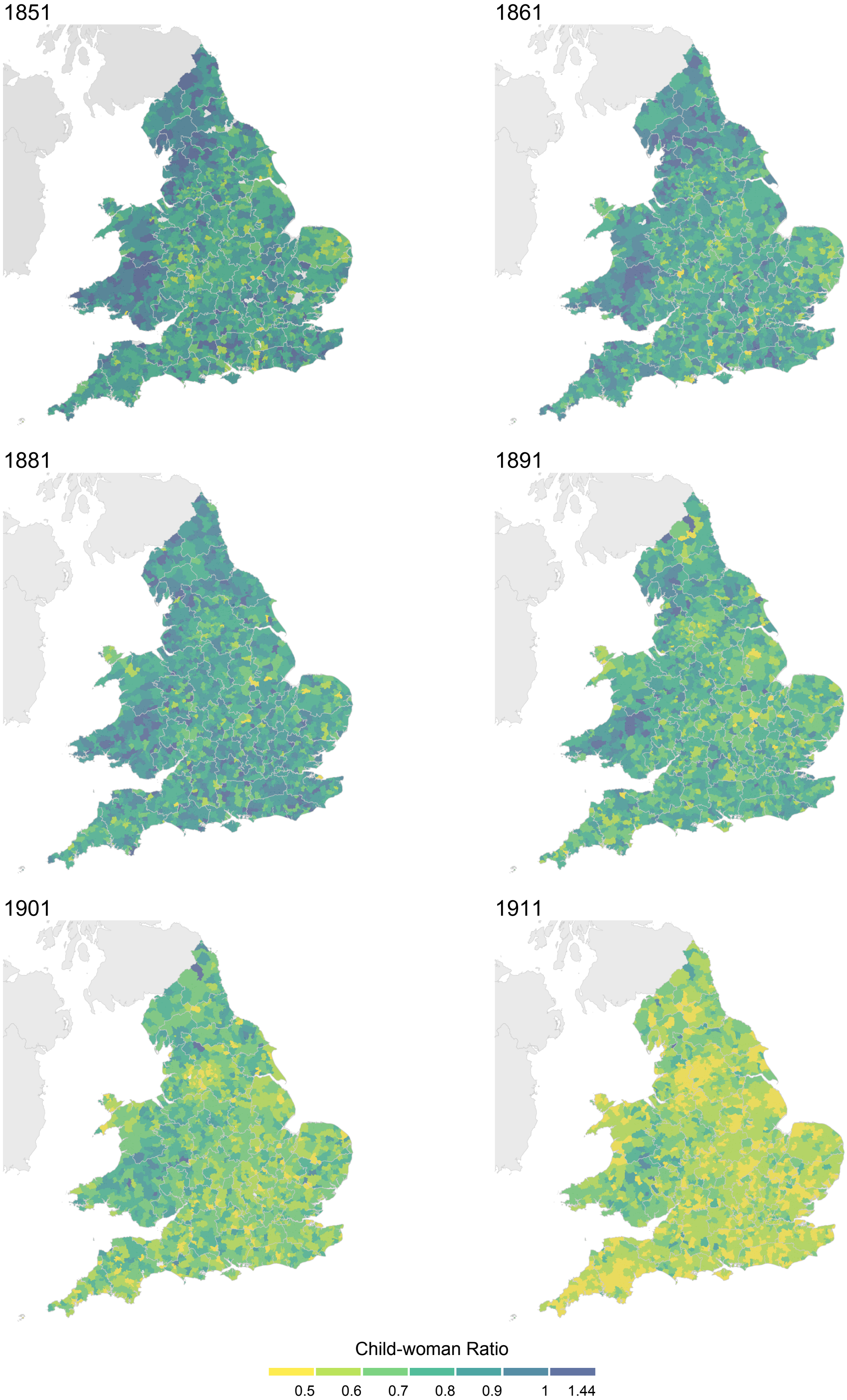

Source: Schürer and Higgs (2014)
Base maps: Registration sub-district boundaries for England and Wales

Fig. A4 Child-woman ratios for skilled manual workers (children aged 0–4 per married spouse present women aged 15–54) in registration sub-districts, England and Wales, 1851–1911

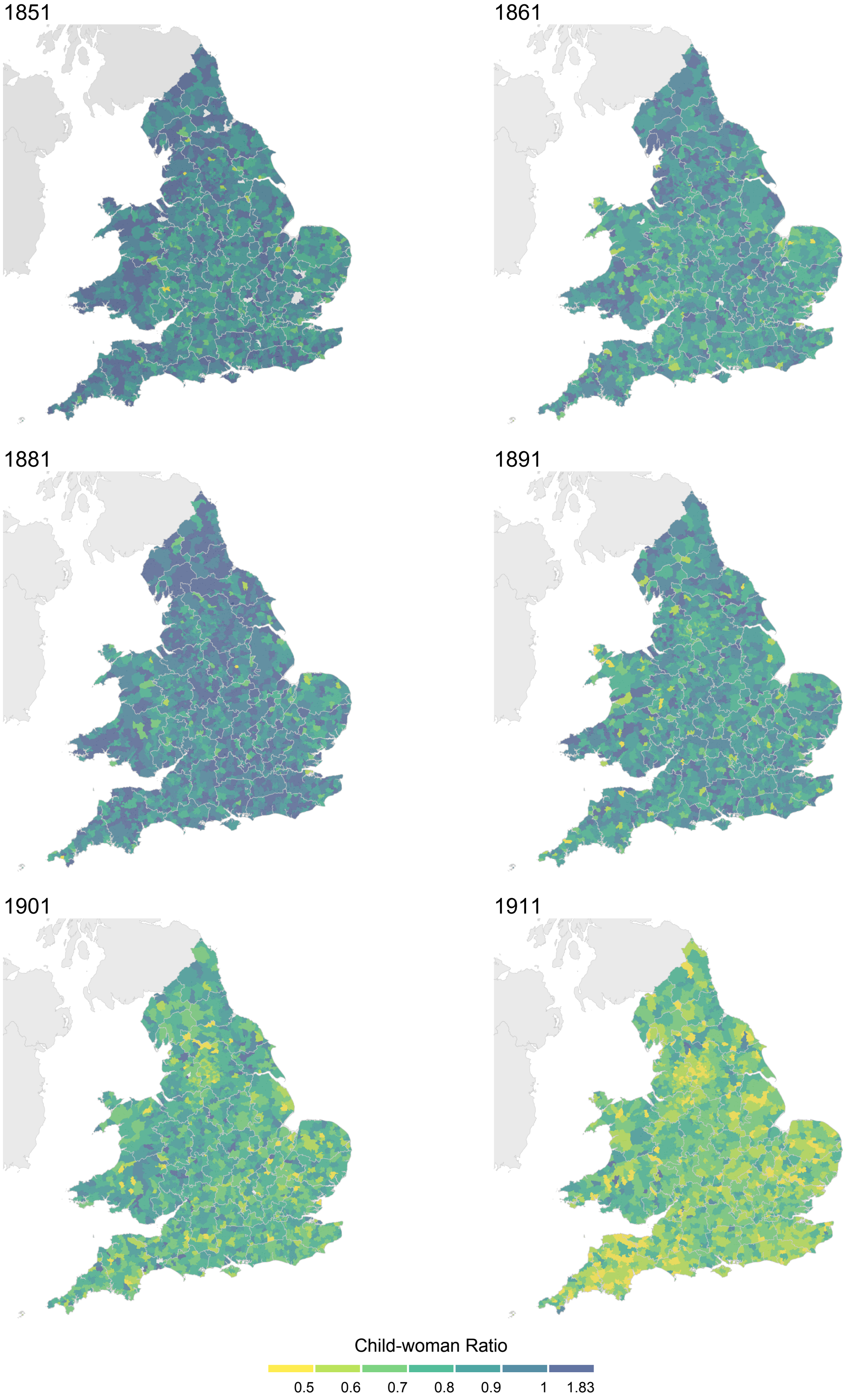

Source: Schürer and Higgs (2014)
Base maps: Registration sub-district boundaries for England and Wales

Fig. A5 Child-woman ratios for semi-skilled manual workers (children aged 0–4 per married spouse present women aged 15–54) in registration sub-districts, England and Wales, 1851–1911

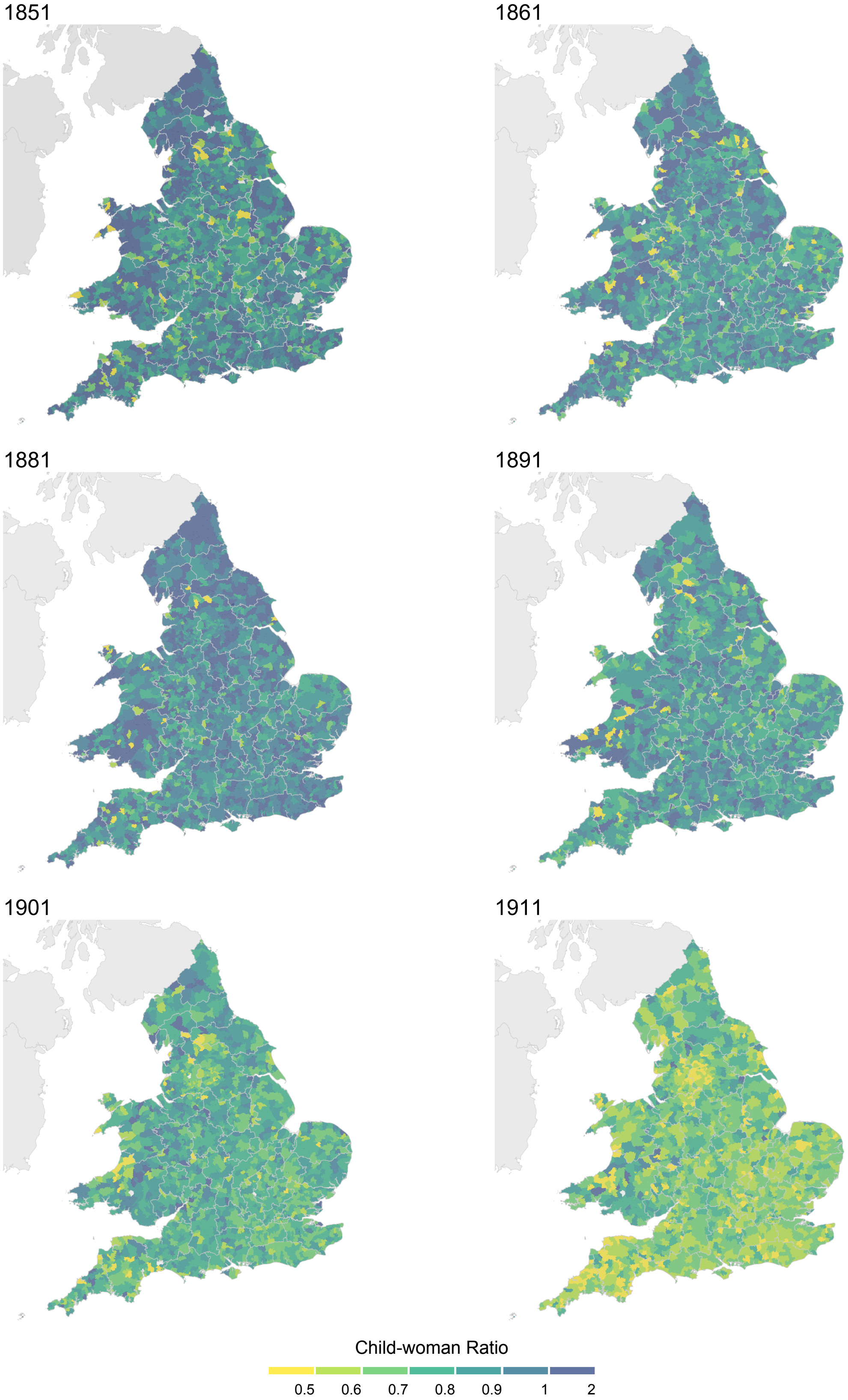

Source: Schürer and Higgs (2014)
Base maps: Registration sub-district boundaries for England and Wales

Fig. A6 Child-woman ratios for unskilled workers (children aged 0–4 per married spouse present women aged 15–54) in registration sub-districts, England and Wales, 1851–1911

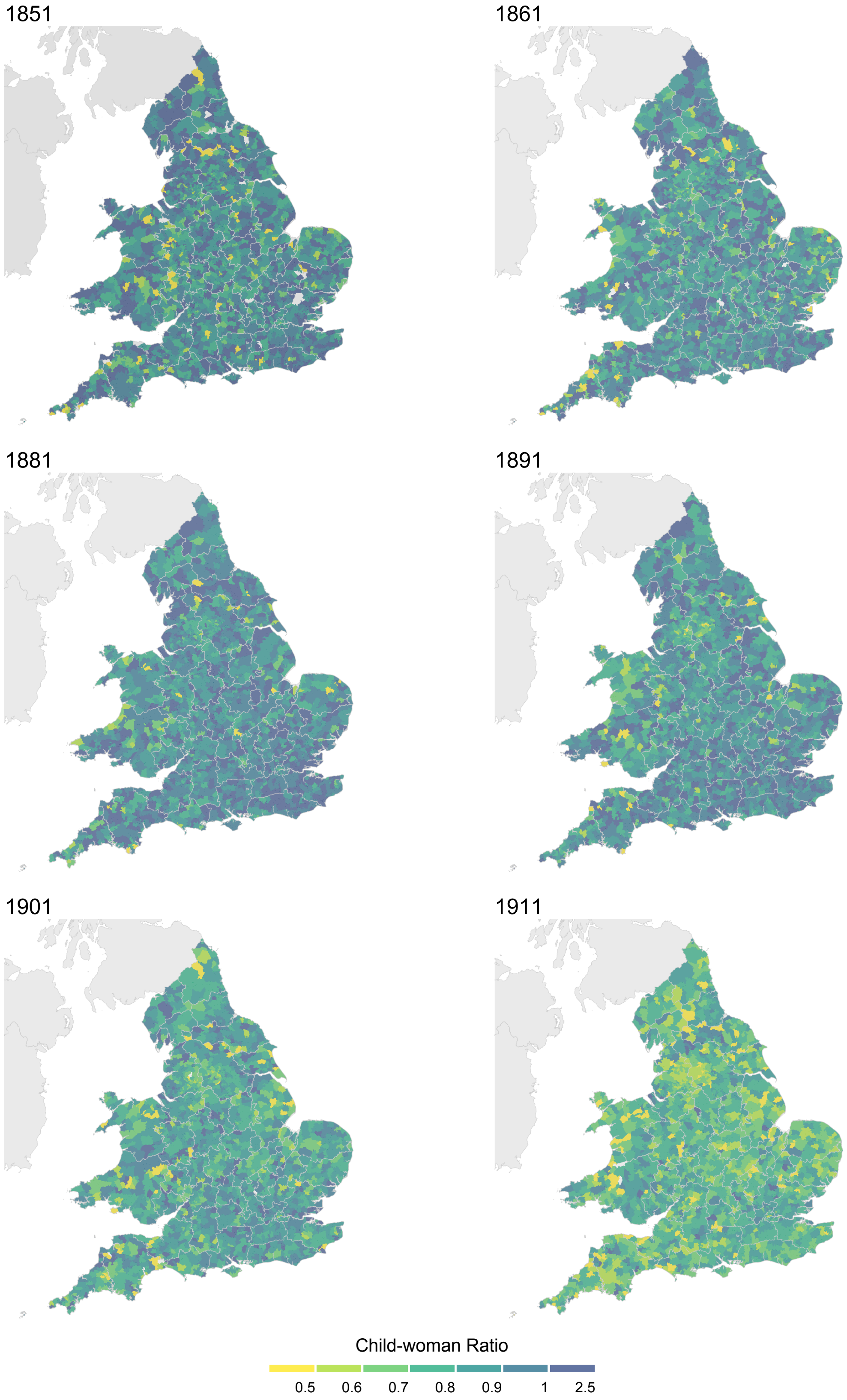

Source: Schürer and Higgs (2014)
Base maps: Registration sub-district boundaries for England and Wales

Fig. A7 Model estimates with and without fixed-effects for the relationship between marital net fertility (number of children aged 0–4) and husband's social class, England and Wales, 1851–1911.

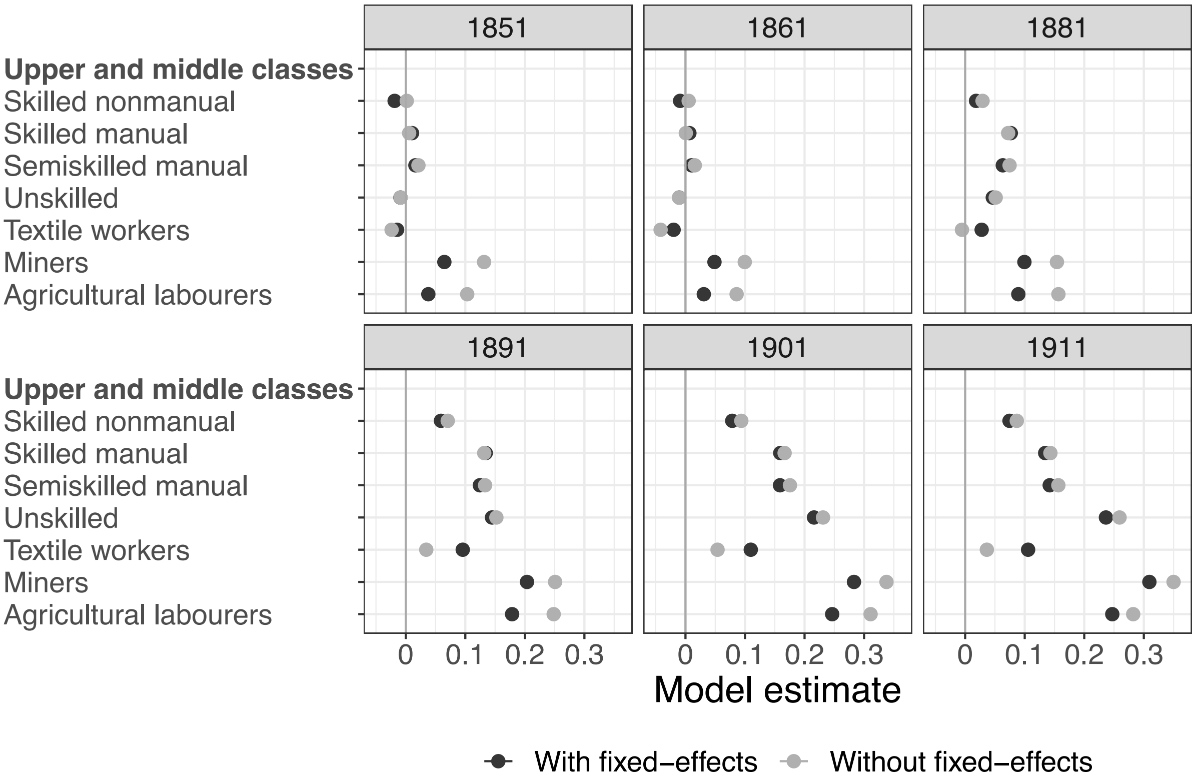

Note: *Models control for age of woman, age difference between spouses, household status, and wife's distance from place of birth.*
Source: Calculated using Schürer and Higgs (2014) and Day (2018b).

Fig. A8 Model estimates with and without fixed-effects for the relationship between marital net fertility (number of children aged 0–4) and wife's lifetime migration, in kilometres (km), England and Wales, 1851–1911.

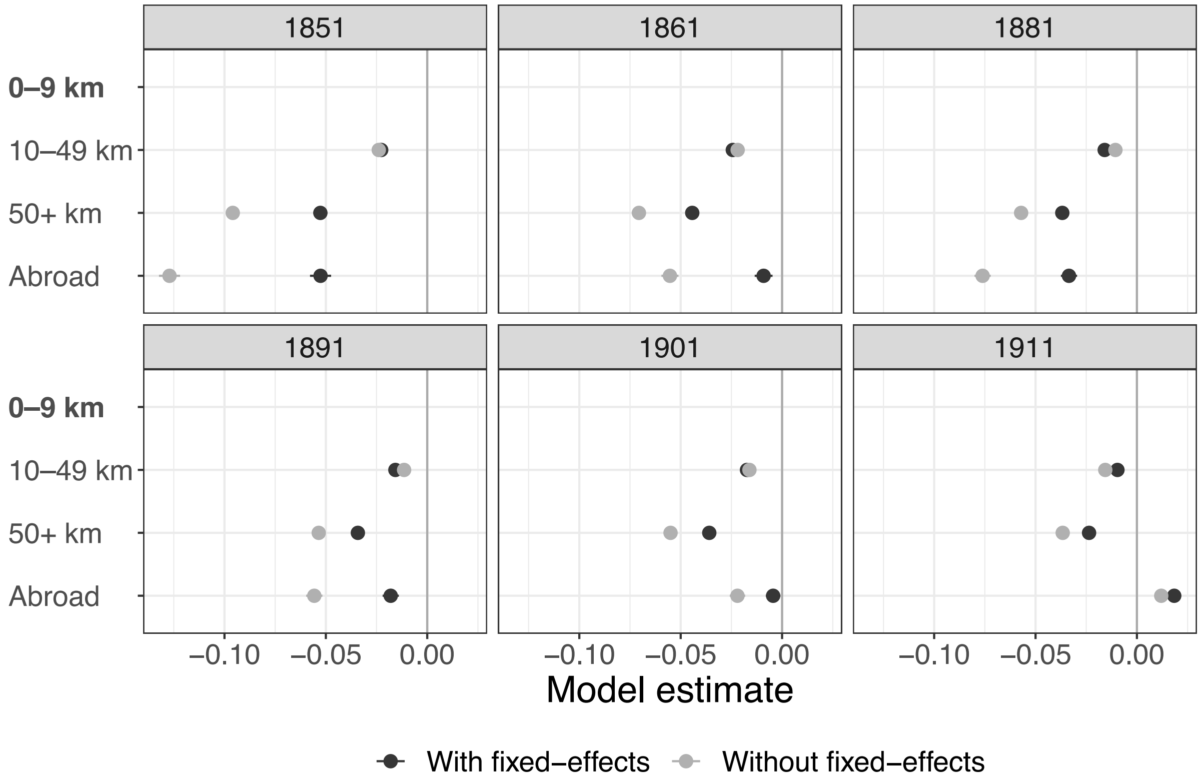

Note: *Models control for age of woman, age difference between spouses, household status, and husband's social class.*
Source: Calculated using Schürer and Higgs (2014) and Day (2018b).

Fig. A9 Model estimates for the relationship between marital net fertility (number of children aged 0–4) and wife's life-time migration by husband's social class, England and Wales, 1851–1911

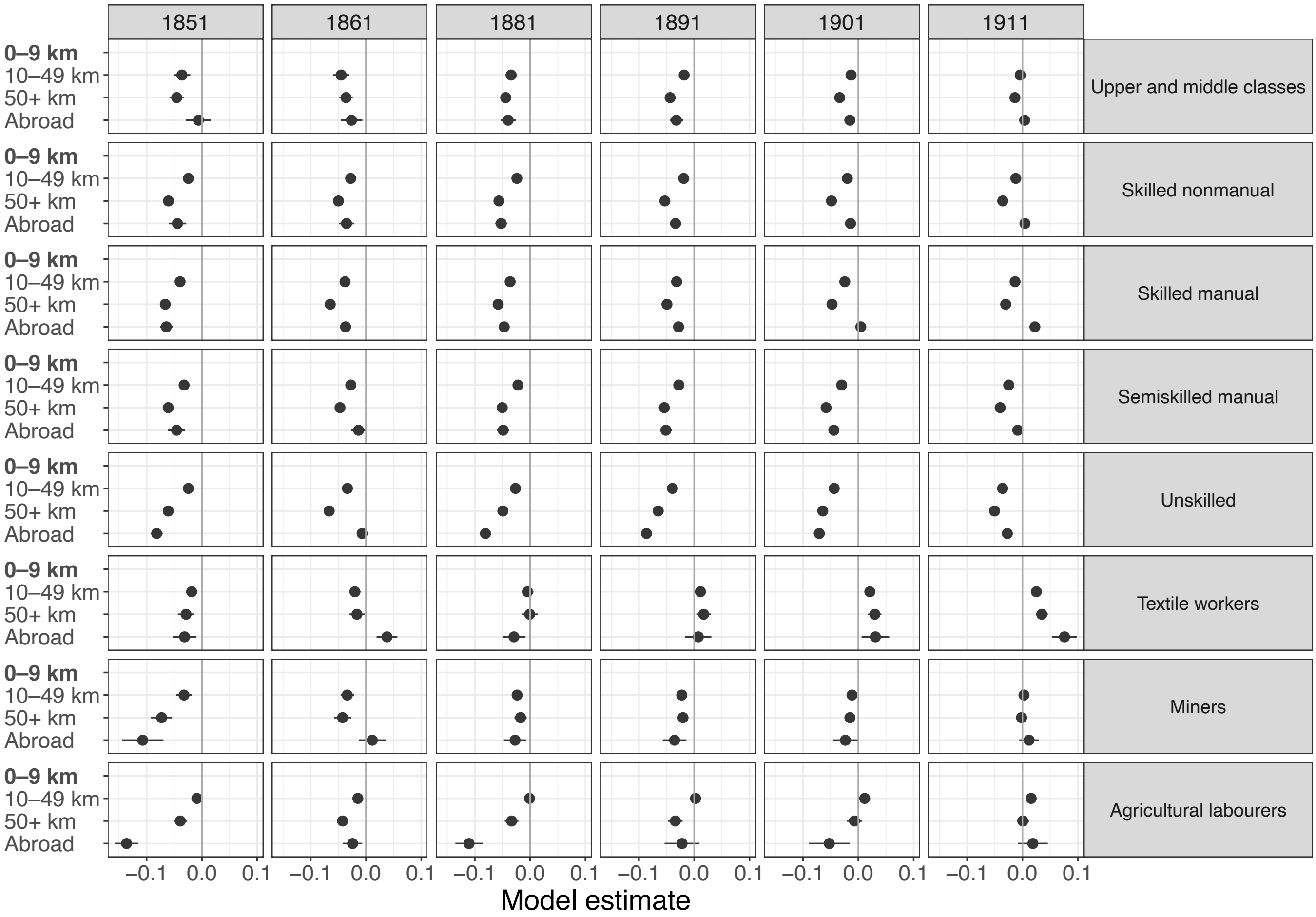

Note: *Models control for age of woman, age difference between spouses, and husband's status within the household.*

Source: Calculated using Schürer and Higgs (2014) and Day (2018b).

Table A1 Distribution of census and study populations across all census years, England and Wales, 1851–1911.

	**1851**	**1861**	**1881**	**1891**	**1901**	**1911**
Full population	17,562,522	19,320,569	26,124,585	29,050,639	32,493,071	36,352,548
Women	8,947,259	9,919,213	13,387,591	14,910,782	16,686,834	18,677,255
Women aged 15–54	4,880,689	5,393,415	7,158,108	8,166,330	9,553,798	10,846,367
Married women aged 15–54	2,520,617	2,862,231	3,769,990	4,164,075	4,845,237	5,616,969
Married women aged 15–54 (with spouse)	2,223,968	2,541,617	3,235,007	3,507,452	4,111,065	4,659,742

Source: Calculated using Schürer and Higgs (2014).

Table A2 Full model estimates for the relationship between marital net fertility (number of children aged 0–4) and our control variables, England and Wales, 1851–1911.

	**1851**		**1861**		**1881**		**1891**		**1901**		**1911**	
	coef	*p*	coef	*p*	coef	*p*	coef	*p*	coef	*p*	coef	*p*
Age Group of Wife												
15–19	-0.766	.000	-0.761	.000	-0.734	.000	-0.736	.000	-0.612	.000	-0.485	.000
20–24	-0.309	.000	-0.293	.000	-0.249	.000	-0.250	.000	-0.183	.000	-0.081	.000
25–29	0.009	.000	0.015	.000	0.061	.000	0.055	.000	0.075	.000	0.104	.000
30–34	Ref.		Ref.		Ref.		Ref.		Ref.		Ref.	
35–39	-0.158	.000	-0.170	.000	-0.220	.000	-0.213	.000	-0.216	.000	-0.213	.000
40–44	-0.508	.000	-0.538	.000	-0.604	.000	-0.575	.000	-0.538	.000	-0.514	.000
45–49	-0.964	.000	-0.975	.000	-1.075	.000	-1.003	.000	-0.911	.000	-0.839	.000
50–54	-1.191	.000	-1.190	.000	-1.268	.000	-1.173	.000	-1.054	.000	-0.959	.000
Age Difference Between Spouses												
Wife older	-0.062	.000	-0.049	.000	-0.053	.000	-0.043	.000	-0.036	.000	-0.014	.000
Husband 0–2 years older	Ref.		Ref.		Ref.		Ref.		Ref.		Ref.	
Husband 3–5 years older	0.004	.005	0.010	.000	0.003	.022	0.002	.128	0.000	.931	-0.003	.002
Husband >6 years older	-0.076	.000	-0.073	.000	-0.091	.000	-0.077	.000	-0.049	.000	-0.033	.000
Husband's Household Position												
Head of household	Ref.		Ref.		Ref.		Ref.		Ref.		Ref.	
Other	-0.422	.000	-0.437	.000	-0.534	.000	-0.429	.000	-0.331	.003	-0.316	.000
Husband's Social Class												
Upper and middle classes	Ref.		Ref.		Ref.		Ref.		Ref.		Ref.	
Skilled nonmanual	-0.019	.000	-0.010	.000	0.017	.000	0.058	.000	0.078	.000	0.074	.000
Skilled manual	0.009	.001	0.005	.058	0.075	.000	0.132	.000	0.158	.000	0.134	.000
Semiskilled manual	0.015	.000	0.009	.001	0.062	.000	0.123	.000	0.158	.000	0.141	.000
Unskilled	-0.010	.001	-0.012	.000	0.045	.000	0.142	.000	0.213	.000	0.235	.000
Textile workers	-0.016	.000	-0.022	.000	0.026	.000	0.093	.000	0.108	.000	0.105	.000
Miners	0.063	.000	0.046	.000	0.097	.000	0.200	.000	0.281	.000	0.308	.000
Agricultural labourers	0.037	.000	0.029	.000	0.086	.000	0.175	.000	0.243	.000	0.245	.000
Unknown	-0.083	.000	-0.031	.000	-0.015	.000	0.039	.000	0.078	.000	0.104	.000
Distance From Wife's Place of Birth												

Less than 10 km	Ref.		Ref.		Ref.		Ref.		Ref.		Ref.	
10–50 km	-0.027	.000	-0.031	.000	-0.026	.000	-0.025	.000	-0.024	.000	-0.015	.000
More than 50 km	-0.060	.000	-0.053	.000	-0.049	.000	-0.051	.000	-0.049	.000	-0.031	.000
Abroad	-0.066	.000	-0.020	.000	-0.057	.000	-0.047	.000	-0.023	.000	0.003	.044
Unknown	-0.077	.000	-0.073	.000	-0.066	.000	-0.044	.000	-0.041	.000	0.034	.030
Adjusted R-squared	0.210		0.206		0.234		0.217		0.205		0.203	
Number of RSDs	2,176		2,189		2,175		2,110		2,060		2,009	
Number of women	2,223,976		2,541,617		3,235,007		3,507,452		4,111,065		4,659,742	

Source: Calculated using Schürer and Higgs (2014) and Day (2018b).

Table A3 Incidence rate ratios (IRR) from Poisson regression models for the relationship between marital net fertility (number of children aged 0–4) and husband's social class and wife's life-time migration, England and Wales, 1851–1911.

	**1851**		**1861**		**1881**		**1891**		**1901**		**1911**	
	IRR	*p*	IRR	*p*	IRR	*p*	IRR	*p*	IRR	*p*	IRR	*p*
Husband's Social Class												
Upper and middle classes	Ref.		Ref.		Ref.		Ref.		Ref.		Ref.	
Skilled nonmanual	0.978	.000	0.990	.005	1.021	.000	1.082	.000	1.139	.000	1.152	.000
Skilled manual	1.011	.005	1.007	.061	1.089	.000	1.189	.000	1.279	.000	1.275	.000
Semiskilled manual	1.018	.000	1.011	.004	1.073	.000	1.175	.000	1.278	.000	1.289	.000
Unskilled	0.988	.003	0.986	.000	1.053	.000	1.202	.000	1.372	.000	1.466	.000
Textile workers	0.980	.000	0.973	.000	1.029	.000	1.135	.000	1.189	.000	1.207	.000
Miners	1.059	.000	1.043	.000	1.106	.000	1.270	.000	1.458	.000	1.571	.000
Agricultural labourers	1.039	.000	1.031	.000	1.104	.000	1.249	.000	1.427	.000	1.497	.000
Unknown	0.872	.000	0.957	.000	0.959	.000	1.024	.000	1.090	.000	1.174	.000
Distance From Wife's Place of Birth												
Less than 10 km	Ref.		Ref.		Ref.		Ref.		Ref.		Ref.	
10–50km	0.968	.000	0.964	.000	0.970	.000	0.969	.000	0.969	.000	0.979	.000
More than 50 km	0.926	.000	0.935	.000	0.942	.000	0.937	.000	0.932	.000	0.950	.000
Abroad	0.920	.000	0.977	.000	0.930	.000	0.940	.000	0.970	.000	1.008	.005
Unknown	0.903	.000	0.910	.000	0.924	.000	0.947	.000	0.946	.000	1.059	.022
Number of RSDs	2,176		2,189		2,175		2,110		2,060		2,009	
Number of women	2,223,976		2,541,617		3,235,007		3,507,452		4,111,065		4,659,742	

Note: Models control for age of woman, age difference between spouses, and household status.
Source: Calculated using Schürer and Higgs (2014) and Day (2018b).
